# Neonatally-derived multipotent Islet-1+ Mesp1+FOXA2+ stem cell clones restore cardiac function in sheep

**DOI:** 10.3389/fcvm.2025.1671367

**Published:** 2026-01-05

**Authors:** Lorelei Hughes, Jonathan Baio, Nahidh Hasaniya, Leonard Bailey, Julia Kim, Danielle Yanez, Edward Austin, Richard Vega, Paola Rivera Morales, Victor Camberos, Christopher G. Wilson, Alicia L. Veliz, Mary Kearns-Jonker

**Affiliations:** 1 Department of Pathology and Human Anatomy, Loma Linda University School of Medicine, Loma Linda, CA, United States; 2 Department of Cardiovascular and Thoracic Surgery, Loma Linda University School of Medicine, Loma Linda, CA, United States; 3 Department of Pediatric Cardiology, Loma Linda University Medical Center, Loma Linda, CA, United States; 4 Lawrence D. Longo MD Center for Perinatal Biology, School of Medicine, Loma Linda University, Loma Linda, CA, United States

**Keywords:** Islet-1, FOXA2, Mesp-1, allogeneic, cardiac repair, stem cell transplantation, transcriptomics, large animal

## Abstract

**Introduction:**

Stem cell therapeutics is an area of active investigation for the treatment of cardiovascular disease. Unlike adults, neonatal hearts possess unique regenerative capacity immediately after birth, suggesting that neonatal cardiovascular tissue may be a promising and untapped resource of stem cells. In the current study, we present the unique transcriptome and differentiation capability of neonatal *ISL1*+ *MESP1*+ *FOXA2*+ stem cell clones isolated from humans. Comparable *ISL1*+ *MESP1*+ *FOXA2*+ stem cell clones were then isolated from sheep for functional analysis in a sheep model of myocardial infarction and allogeneic stem cell-based repair without immunosuppression.

**Methods:**

The transcriptome of early-stage, human neonatal ISL1+ stem cell clones was identified by RNAseq analysis. Differentiation capability was validated by flow cytometry, RT-qPCR and electrophysiology. Matched ISL1+ neonatal sheep stem cell clones were isolated for the purpose of developing an allogeneic, preclinical large animal model of ISL1+ stem cell-based repair in sheep. A myocardial infarction was induced by ligation of left anterior descending coronary artery followed by ISL1+ stem cell transplantation three-four weeks later by direct intracardiac injection in the absence of immunosuppression. The *in vivo* transplant outcomes in stem cell-treated vs. controls were assessed at three months after myocardial infarction by echocardiography, immunohistochemistry, western blot, RT-qPCR, and RNAseq analyses.

**Results:**

Neonatally-derived ISL1+ clones restored cardiac function to normal levels as shown by echocardiography. Stem cell retention was identified by histology in the cardiovascular repair zone and transcriptomic analysis identified the contribution of several signaling pathways leading to activation of paracrine and cardiogenic effects in the stem-cell treated regions of the heart. We further define the contribution of immunosuppressive mediators that contribute to stem cell retention and factors that stimulate endothelial cell recruitment in this allogeneic model of stem cell-based therapy.

**Conclusion:**

*ISL1*+ *MESP1*+ *FOXA2*+ stem cell clones isolated from neonatal cardiovascular tissue represent a novel resource of cells with the capacity to restore cardiac function following myocardial infarction in a preclinical large animal model.

## 1 Introduction

Stem cell-based therapies have the potential to facilitate cardiovascular regeneration after myocardial infarction (MI), yet the ability to identify the optimal stem cell source, differentiation stage and method of administration necessary to achieve stable functional improvement in the absence of arrhythmias has been challenging in large animal models. Restoration of cardiac function and significant retention has recently been shown in non-human primates and pigs using fully differentiated cardiomyocytes derived from both induced pluripotent stem cells (iPSCs) and human embryonic stem cells (hESCs), however, the onset of arrhythmias in several of these models has raised safety concerns which have limited advancement of these promising findings to the clinic (1–6).

One of the advantages of using hESCs or iPSCs as a broad resource for stem cell-based therapy is their ability to differentiate into both mesodermal or endodermal derivatives as needed for multiple applications. The mesendoderm gives rise to most transplanted organs including, liver, kidney, lung, pancreas, intestine, and heart. Isolation and banking of cells at this early stage has the potential to minimize the number of patients waiting for organ transplants if stem cell-based treatments can be developed with the ability to recover function. Transcription factors expressed just prior to lineage-specific commitment facilitate isolation and purification of early-stage stem cells. ISL1, for example, is a transcription factor which is required for embryonic cardiogenesis (7–9) and has been used to purify early-stage hESCs for cardiac repair in recent clinical trials (10). Lineage tracing studies have provided evidence to show that early-stage ISL1+ stem cells contribute to both first and second heart fields (7–9). Although ISL1+ cells have a well-documented ability to improve cardiac function in small animal models, their presence is significantly reduced in the heart after the neonatal period. In the current study, we isolated, cloned and characterized the functional potential of ISL1+ stem cells from neonatal cardiovascular tissue. Neonatal hearts possess a well-documented regenerative capacity for a short period of time immediately after birth, yet neonatal human cardiovascular tissue has not been tapped as a resource of stem cells for cardiovascular repair and have not been previously tested in large animal models.

Subpopulations of ISL1+ cells which co-express the transcription­­ factor MESP1, a master regulator of mesendoderm formation and cardiac development, are of particular interest (11). MESP1 is expressed transiently at the earliest stages of cardiovascular lineage commitment but has also been identified in progenitors capable of differentiation into skeletal muscle and bone (12). The transcription factor FOXA2 is present in both mesoderm and endoderm committed progenitors and is expressed in cardiovascular progenitor cells which can differentiate into both ventricles of the heart and the epicardium (13). Notably, the extracellular matrix and tissue-specific environment play a key role in the determination of lineage specificity as these cells differentiate. In the cardiovascular repair model, we hypothesize that an early-stage *ISL1+ FOXA2+ MESP1*+ stem cell would be advantageous given the ability of this cell to give rise to all components of the heart while retaining multilineage differentiation capability when cultured in lineage-specific differentiation conditions, if needed for other clinical applications. The availability of clonal populations of these cells represents a potentially valuable stem cell resource.

In this report, we present the transcriptome of several subpopulations of ISL1+ stem cell clones which were found to exist transiently in the cardiovascular tissue of human neonates. Although the enhanced regenerative ability of the neonatal heart is well-established, details defining the transcriptome, differentiation capacity, and functionality of early-stage stem cells isolated from human neonates is lacking. We show that select early-stage *ISL1+ MESP1+ FOXA2+* clones possess the capacity to differentiate into multiple cardiovascular cell types and exhibit an enhanced stemness profile. A matched population of cloned *ISL1+ MESP1+ FOXA2+* cells isolated from neonatal sheep was utilized for functional studies *in vivo* in an allogeneic ovine model of MI without immunosuppression. *ISL1+ MESP1+ FOXA2+* cell transplantation induced significant functional improvement via activation of *YAP1*, *ARGN*, and by the recruitment of host-derived endothelial cells which function to enhance vascularization at the cardiovascular repair site. Donor cell retention and immunosuppression at the local level was achieved, in part, by the expression of Prostaglandin E2 in donor-derived ISL1+ cells.

ISL1+ stage stem cells can be readily isolated and rapidly expanded as clones from patient-derived tissue during the pro-regenerative phase of the neonatal period. The clonal nature of these cells endows them with a high degree of reproducibility. Although cells at this early stage can be isolated from hESCs or iPSCs, the process of purification and expansion is costly and time-consuming. Neonatal stem cells represent a novel alternative with regenerative therapeutic potential.

## 2 Materials and methods

### 2.1 Ethics approval for human and ovine experiments

The Institutional Review Board of Loma Linda University approved protocol (IRB# 5110115) for use of tissue discarded during cardiovascular surgery, without identifiable private information, for this study with a waiver of informed consent. All experimental procedures involving animals were performed within the regulations of the Animal Welfare Act, the National Institutes of Health Guide for the Care and Use of Laboratory Animals, the Panel on Euthanasia of the American Veterinary Medical Association and were approved by the Institutional Animal Care and Use Committee of Loma Linda University (IACUC approval #8130017 and #8110004).

### 2.2 Isolation of early-stage neonatal human and ovine stem cells

Neonatal human (1 day—1 month) and ovine (9–10 days old) stem cells were previously isolated and maintained as described in Fuentes et al. and Hou et al. (14, 15). Human ISL1+ cardiovascular stem cell clones were isolated from cardiac tissue which was discarded during cardiovascular surgeries that were necessary for the health and well-being of the patients. Only tissue that needed to be removed as a part of these procedures and would otherwise be discarded was collected. Ovine cardiovascular stem cell clones were isolated from atrial cardiac tissue. Tissue from both sources was cut into ∼1.0 mm^3^ clumps, enzymatically digested using collagenase (Roche, Indianapolis, IN), and passed through a 40-mm cell strainer. Cells were cloned by limiting dilution. Clones were expanded and screened via flow cytometry for co-expression of ISL1+ and CKIT. Clones were expanded in growth media which included 66% Medium 199 (Life Technologies, Carlsbad, CA), either 10% fetal bovine serum for human stem cells (HyClone, Logan, UT) or 10% lamb serum for ovine stem cells (Life Technologies, Carlsbad, CA), 22% endothelial cell growth media (Lonza, Basel, Switzerland), 100 µg/mL penicillin-streptomycin (Life Technologies, Carlsbad, CA), and 1.0% minimum essential medium non-essential amino acids solution (Life Technologies, Carlsbad, CA).

### 2.3 Multi-color flow cytometry for detection of early-stage markers

Stem cell populations were fluorescently labeled with antibodies using manufacturer-recommended concentrations. Stem cells were stained for PDGFRα, CKIT, CXCR4, and SSEA1, fixed in 4% PFA (Sigma Aldrich, St. Louis, MO), permeabilized in 0.1% Tween-20 (Sigma Aldrich, St. Louis, MO), blocked in 0.6 M glycine (Sigma Aldrich, St. Louis, MO) containing 10% BSA (Research Products International Corp, Mt. Prospect, IL), and stained for intracellular transcription factors (ISL1 and MESP1). Stained stem cells were analyzed using a MACSQuant® analyzer (Miltenyi Biotec, Auburn, CA). Quantification of data was performed using FlowJo software version 10 (Ashland, OR). Compensation was performed using UltraComp eBeads (Life Technologies, Grand Island, NY). Antibodies are described in Supplementary Table 1.

### 2.4 Carboxyfluorescein succinimidyl ester (CFSE) labeling

Within 24 h of transplantation, neonatal ovine stem cells were labeled with CFSE. Briefly, 1 mL of CFSE (BioLegend, San Diego, CA) at a concentration of 5 µM in DPBS was added to 1 mL DPBS containing 10 × 10^6^ stem cells for 10 min at 37 °C. Labeling was confirmed by flow cytometry prior to transplantation.

### 2.5 Myocardial infarction and stem cell transplantation

The isolation of sheep ISL1+ stem cell clones was reported previously (15). Two 9–10 day old neonatal Western sheep (Nebeker Ranch, Lancaster, CA) were anesthetized with thiopental sodium (10 mg.kg^−1^, IV) and anesthesia was maintained with inhalation of 1.5%–3% isoflurane. Tissue from the right atrium was obtained under a shared tissue protocol. Euthanasia was performed under general anesthesia with an overdose of Euthasol (pentobarbital sodium 100 mg.kg^−1^ and phenytoin sodium 10 mg.kg^−1^; Virbac, Ft. Worth, TX) administered intravenously.

Ten additional female Western sheep ≤1 year of age (Nebeker Ranch) weighing 50 ± 10 kg were used for the ovine model of MI. Only Q fever negative sheep were included. A power analysis was used to determine the number of animals used within the study. Animals were randomly assigned to either the control or experimental group. Ewes were housed in groups of two or more within the animal care facility of Loma Linda University Medical Center under a 12 h dark/light cycle with access to food *ad libitum*. Sheep were sedated with 0.5 mg/Kg midazolam iv. Telazol (4 mg/kg IV to effect) was given for induction and endotracheal intubation was performed. Maintenance was accomplished with 1.5%–3% isoflurane in 100% oxygen, delivered via ventilator. Monitoring included invasive blood pressure (auricular artery), end tidal CO2, oximetry, and core body temperature. The heart was exposed by left thoracotomy and the left anterior descending coronary artery was permanently ligated at the midpoint between the apex and the base. Post-surgery analgesia was provided by Buprenorphine (0.005–0.01 mg/kg) IM q4 h for a period of 72 h. The animals were monitored after surgery for signs of fever and pain using the Sheep Grimace Scale, nutritional deficiency and weight loss. Three-four weeks post-infarction, a second surgery took place to administer the stem cells for cardiovascular repair, administered by intramyocardial injection. Ten million CFSE-labeled stem cell clones isolated from 9-day old ovine neonates were resuspended in 1 mL of sterile PBS. Ten injections containing one million cells each were administered directly in the periphery of the infarct. For the second surgery, sedation, maintenance, induction of anesthesia, and analgesia was achieved using the same drugs as described in the first surgery, at the same doses. Post-surgical monitoring was done as described after the first surgery. After two months, the ewes were sacrificed and the infarcted and non-infarcted regions of the hearts were mapped and sectioned into pieces, then snap frozen in liquid nitrogen. Euthanasia was performed under general anesthesia as described above. Echocardiograms and EKGs were performed at the time of surgery and at the time of euthanasia.

### 2.6 Immunohistochemistry, imaging, and analysis

Antibodies and conditions used for immunohistochemistry (IHC) analysis are indicated in Supplementary Table 2. Frozen tissue was sectioned using a Leica CM1900 cryostat (Leica Biosytems, Buffalo Grove, IL). Slides undergoing staining for KI67, ISL1, TROPI, VWF, and CD14 were fixed, blocked and stained. Sections were mounted using Prolong Gold with DAPI (Life Technologies, Eugene, OR). Stained slides were imaged using a LSM 710 NLO laser-scanning, confocal microscope (Carl Zeiss Microscopy GmbH, Jena, Germany). Image-Pro Plus (Media Cybernetics, Rockville, MD) was used for quantification.

### 2.7 RNA purification

RNA was isolated from neonatal human stem cell clones placed in QIAzol (Qiagen, Valencia, CA) or TRIzol (Life Technologies, Carlsbad, CA). RNA was purified from ovine tissue samples using the miRNeasy or RNeasy Fibrous Tissue Mini Kit (Qiagen, Valencia, CA). cDNA was prepared using 1–2 µg of RNA and Superscript III (Life Technologies, Carlsbad, CA) for RT-qPCR experiments.

### 2.8 Quantitative reverse transcription polymerase chain reaction (RT-qPCR)

RT-qPCR was performed using iTaq™ Universal SYBR® Green Supermix (Bio-Rad, Hercules, CA) and was run using a Bio-Rad CFX96 Touch Real-Time PCR Detection System (Bio-Rad) or an iCycler iQ™5 PCR Thermal Cycler (Bio-Rad, Hercules, CA). The plates were run using the following conditions: 94 °C for 10 min, 94 °C for 15 s, 56–60 °C for 1 min, 72 °C for 30 s, repeated for 45 cycles. RT-qPCR products were visualized using gel electrophoresis. Primers were designed using the National Center for Biotechnology Information Primer-BLAST system and were manufactured by Integrated DNA Technologies (Coralville, IA). Primer sequences are listed in Supplementary Table 3.

### 2.9 Western blot with protein simple

For Akt signaling activation analysis, protein was isolated from ovine cardiac tissue. Protein concentrations were determined using the Pierce Micro BCA Protein Assay Kit (Thermo Scientific, Rockford, IL). A capillary-based western blotting system (ProteinSimple Wes, San Jose, CA) was used to assess protein expression. Data was analyzed with Compass Software (ProteinSimple, San Jose, CA). Prism was used for further analysis. Antibodies and conditions used are listed in Supplementary Table 4.

### 2.10 RNA sequencing of human neonatal ISL1+ stem cell clones and ovine tissue

RNA samples isolated from neonatal ISL1+ stem cell clones were sent to PrimBio Research Institute for RNA-Sequencing (Exton, PA, USA). The analysis was performed as previously described (16). RNA samples isolated from sheep tissue (*n* = 9) including biological and technical replicates were sent to LC Sciences (Houston, TX) for PolyA RNA sequencing analyses. Specifically, the stem cell-treated zones of three sheep were compared against the non-infarct region of the same sheep to investigate specific regional changes in the cell treated zone while accounting for biological variation. The Poly(A) RNA sequencing library was generated following Illumina's TruSeq-stranded-mRNA sample preparation protocol. Quality control analysis and quantification were performed via the Agilent Technologies 2100 Bioanalyzer High Sensitivity DNA Chip. The paired-ended sequencing was performed on Illumina's NovaSeq 6000 sequencing system. Cutadapt and perl scripts were used to remove adapter contaminated reads along with undetermined and low-quality bases. HISAT2 was used to map the reads to the reference genome. The resulting reads were converted to BAM files and sorted using Samtools and assembled via StringTie. All resulting transcriptomes were merged via StringTie and abundances were re-estimated. R package DESeq2 was used to perform differential expression analysis.

### 2.11 Statistical analysis

Normality of data distribution was determined via Shapiro–Wilk test or d'Agostine Pearson omnibus normality test. For protein and transcript expression analysis, either Student's *t*-test or Mann–Whitney *U* test was used to compare the mean of normally or non-normally distributed data, respectively. A two-way ANOVA was performed to measure the effect of stem cell transplantation on cardiac functional measures within and across groups. All data is reported as mean ± standard error. Versions 7 and 8 of Graphpad Prism (GraphPad, La Jolla, CA) were used for statistical analyses. *P* values < 0.05 were assumed to indicate statistical significance.

## 3 Results

### 3.1 The transcriptome of human ISL1+ neonatal stem cells

**Figure 1:**
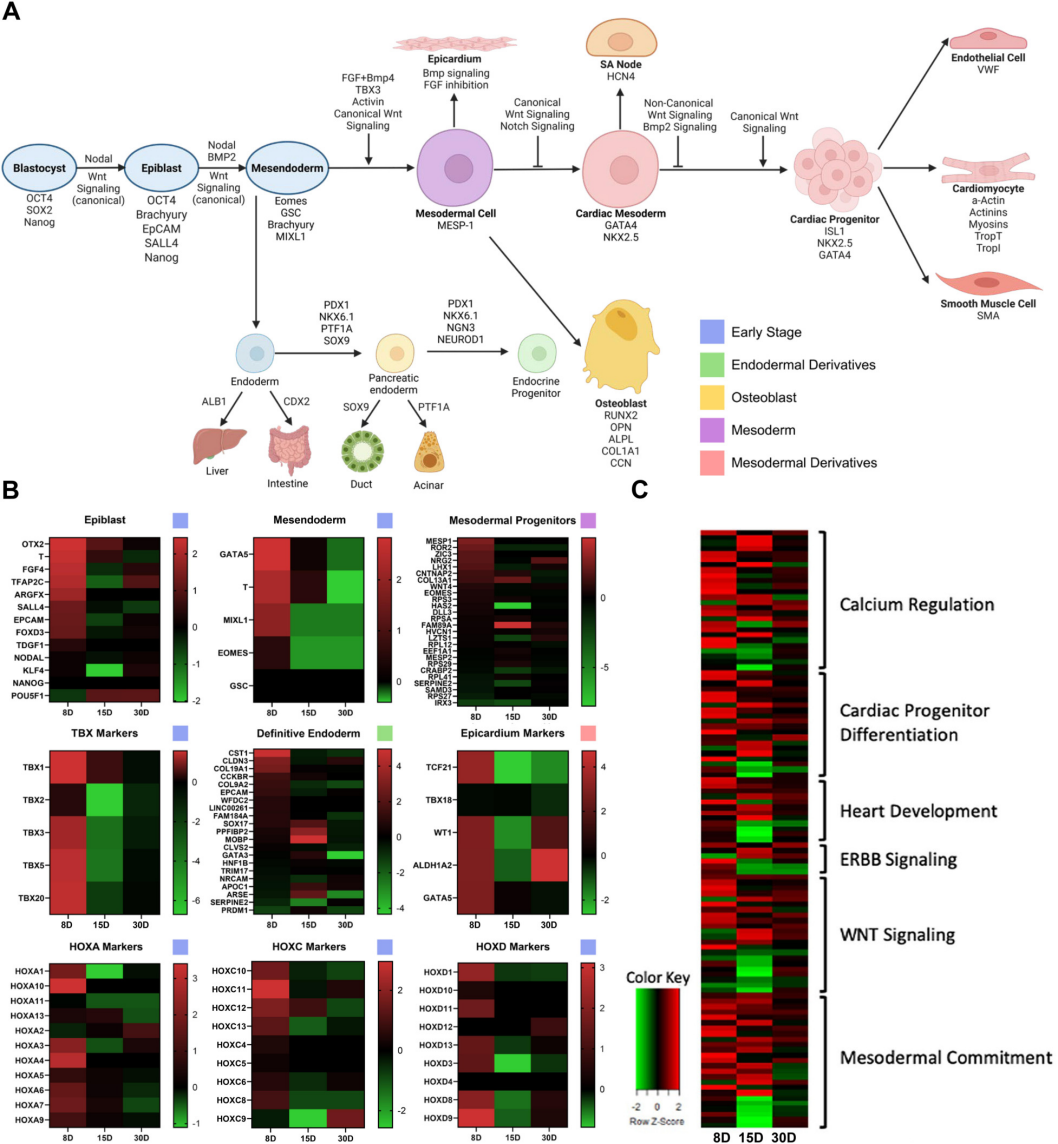
The transcriptome of three ISL1+ stem cell clones. iPSC and hESC lineage tracing studies have established a generally accepted path of differentiation from epiblast to cardiac commitment as shown. Image created with Biorender.com. **(A)** Heatmaps generated with transcriptomic data from three ISL1+ stem cell clones isolated from 8 day old (8D), 15 day old (15D), and 30 day old (30D) neonates demonstrate that the 8D neonatal clone possesses high level expression of early-stage developmental markers, along with epicardial, TBX, and HOX markers. **(B)** Transcripts associated with specific pathways, including cardiac development and differentiation, were elevated in neonatal ISL1+ stem cell clones **(C)**.

Conventional differentiation studies have allowed for the determination of the pathway by which stem cells differentiate into mesendodermal derivatives including cardiac lineages (Figure 1A). To determine the stage at which our stem cells most closely align, the unique transcriptome of ISL1+ cells isolated from neonatal cardiac tissue was identified using RNAseq. The transcriptome of the ISL1+ stem cell clone isolated from an 8-day (8D) old neonate is clearly distinguishable from the transcriptome of the clones which were isolated from the 15-day (15D) and 30-day (30D) neonates. The transcriptome of the ISL1+ clone isolated from the 8D old neonate demonstrates enriched expression of early-stage stemness and development markers, including but not limited to, transcripts associated with epiblast and mesendodermal stages of development (Figure 1B) (7, 17–22). Early-stage *ISL1+ MESP+* *FOXA2*+ clones were successfully differentiated into cardiomyocytes, smooth muscle cells, endothelial cells and sinoatrial node following previously established protocols (Supplementary Table 5, Supplementary Figure 1) (23–26). A comparison of our transcriptomic data with Loh et al.'s mesodermal differentiation study suggests that the 8-day neonatal clone exhibited an expression profile most consistent with day 1–2 of differentiation (Supplementary Figure 2) (17). Furthermore, the expression of early-stage transient markers such as *MIXL1*, *MESP1*, *T*, and *TFAP2C* suggests that these cells associate with a pre-mesendodermal stage (8, 16, 27). Neonatal ISL1+ stem cells expressed transcripts related to heart development, ERBB signaling, and Wnt signaling at relatively high levels as demonstrated in Figure 1C (Supplementary Table 6). Clones isolated shortly after birth, during the pro-regenerative window, were selected for further study.

### 3.2 Neonatal human and ovine stem cells express transcripts associated with pluripotency and early development

**Figure 2:**
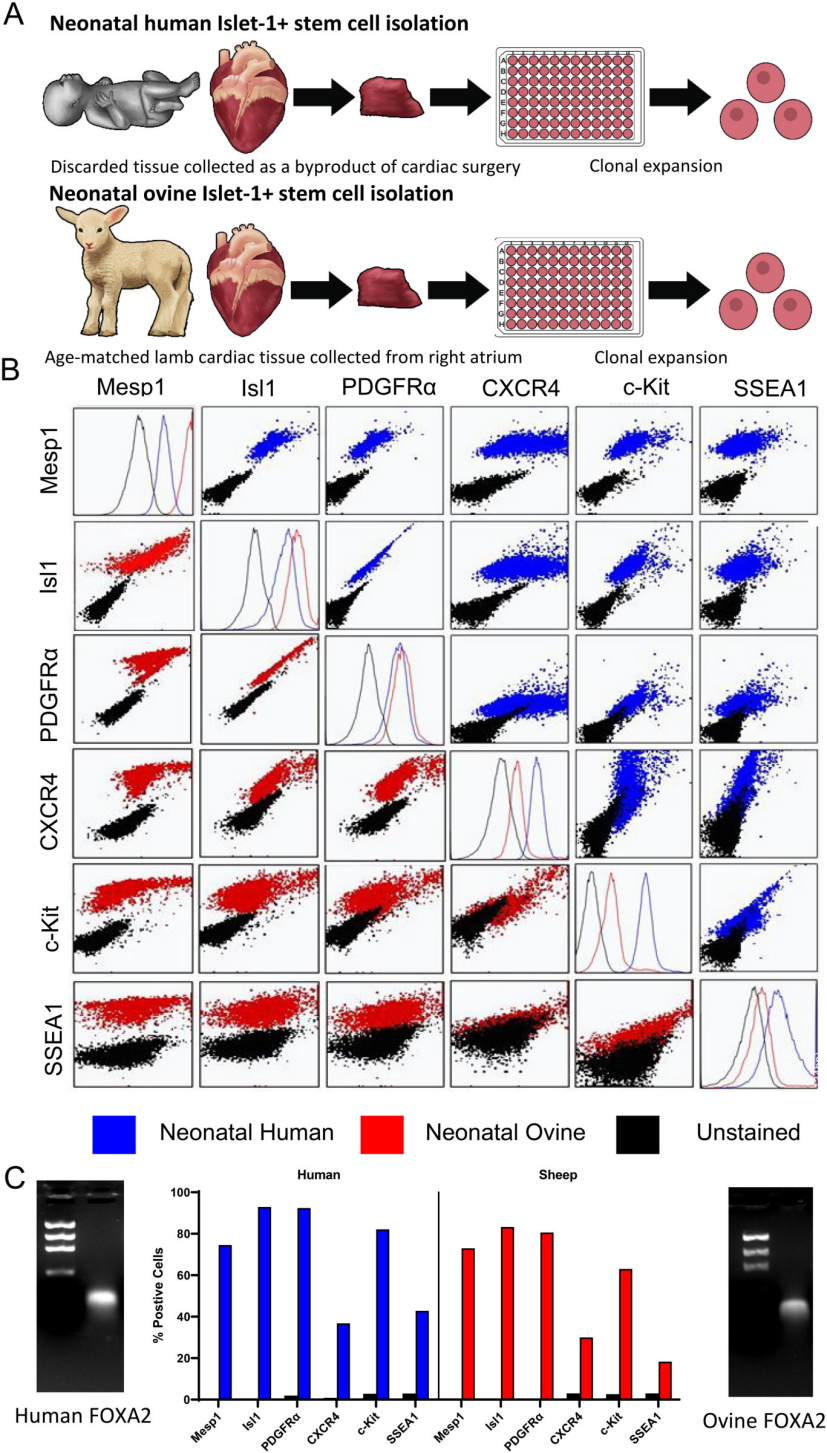
Neonatal human and ovine stem cells express pre-mesodermal markers. Model of human and ovine stem cell isolation. **(A)** Flow cytometry was used to assess the co-expression of early mesodermal developmental markers in neonatal human (blue) and ovine (red) stem cells. **(B)** PCR was used to demonstrate FOXA2 expression in neonatal human and ovine stem cells, one representative clone of each is shown, *n* = 8 total. The percentage of cells expressing each marker identified by flow cytometry comparing ovine and human stem cell clones is shown **(C)**.

To functionally test an equivalent stem cell population in an allogeneic large animal model, comparable early-stage ISL1+ clones were isolated from the ovine right atrium of neonatal sheep at 9–10 days postpartum (Figure 2A). Multicolor flow cytometry was used to compare these cells with those isolated from human neonates (Figure 2B). ISL1, CXCR4, SSEA1, CKIT, MESP1 and PDGFR*α* were co-expressed on cells at this stage in both sheep and human neonatal stem cells. Please refer to Supplementary Tables 7–10 for isotype analysis and further flow cytometry statistical data. *FOXA2* was expressed in sheep and human ISL1+ stem cells as shown by PCR (Figure 2C). Collectively, these results demonstrated a significant overlap in numerous stemness and staging markers. This method of single cell clonal expansion and subpopulation selection provided ovine stem cells which were comparable to those found in humans.

### 3.3 Cell retention and survival in the cardiovascular repair zone

**Figure 3:**
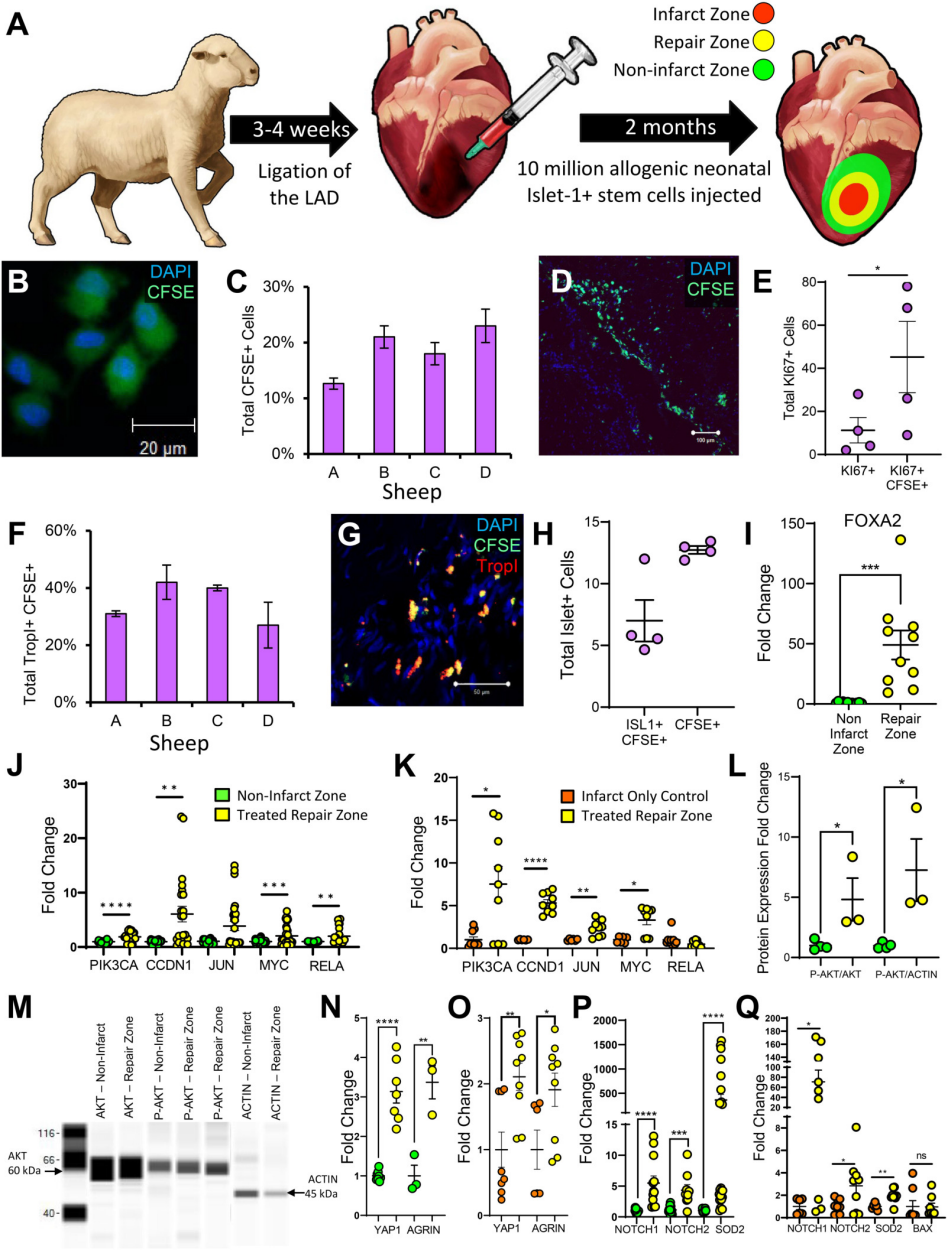
ISL1+ stem cells proliferate in the cardiovascular repair zone and a proportion differentiate into cardiomyocytes and endothelial cells. Sheep myocardial infarction and allogeneic ISL1+ MESP1+ FOXA2+ stem cell transplantation model **(A)** CFSE and DAPI stained ISL1+ clones **(B)** were directly injected peripherally around the infarct. After two months, IHC was performed and stained sections were analyzed using Image-Pro Plus. The percentage of CFSE-stained, transplanted stem cells per sheep is shown graphically in **(C)** and a stained section identifying CFSE and DAPI stained cells is shown in **(D)**. Cells labeled with KI67 **(E)** and TROPI **(F,G)** were quantified by immunostaining. ISL1 was expressed in cells **(H)** located within the cardiovascular repair zone. FOXA2 transcripts **(I)** and transcripts associated with AKT signaling **(J,K)** were elevated within the repair zone (yellow) when compared against both the non-infarct zone (green) and the infarct-only control sheep (orange) as demonstrated by RT-qPCR. Western blotting was performed to confirm AKT activation **(L,M)**. Downstream of AKT signaling, YAP1 and AGRN were elevated within the repair zone (yellow) when compared against the non-infarct zone (green) **(N)** and the infarct-only control (orange) **(O)** NOTCH1/2 and SOD2 were elevated when compared against all controls **(P,Q)**. Additionally, BAX, an apoptosis marker was not elevated when compared with infarct-only controls **(Q)**. These transcript levels were measured by RT-qPCR. Normality of data distribution was determined via the Shapiro–Wilk test or d'Agostine Pearson omnibus normality test (when appropriate). A Student's t-test or Mann–Whitney U test was used to compare the mean of normally or non-normally distributed data, respectively. Immunofluorescence data is presented as the mean ± SEM, *n* = 3–4 ewes per group, each measured in triplicate. RT-PCR and Western Blot data is presented as mean ± SEM, *n* = 1–4 ewes per group, each measured in triplicate.

To test the regenerative capacity of these *ISL1+ MESP1+ FOXA2+* ovine stem cells, a large-animal model of MI and allogeneic cell-based repair was designed (Figure 3A). An MI was induced in experimental sheep via left anterior descending coronary artery (LAD) ligation followed by direct injection of 10 million neonatal ovine *ISL1*+ *MESP1*+ *FOXA2*+ stem cells in the peri-infarct zone 3–4 weeks later. Control sheep also received an MI but did not receive cell treatment. Three months post-infarct, hearts were excised from both groups and sectioned for further analysis. Three zones of interest within the cell-transplanted ovine heart were identified based on visual, histological, and transcriptomic analysis. The infarct zone is the region wherein there is still residual evidence of the initial MI and scarring (Supplementary Figure 3). The repair zone is the border region of the infarct zone wherein the stem cells were transplanted, and the non-infarcted zone is represented by regions of the left ventricle outside of the cell-injected region. Tissue was isolated from each of these zones for subsequent analysis. Prior to transplantation, neonatal ovine stem cells were tagged with Carboxyfluoroscein Succinimidyl Ester (CFSE) to quantify the presence of ISL1+ stem cells at the time of sacrifice. CFSE labeling was validated via fluorescent microscopy and flow cytometry immediately prior to cell injection (Figure 3B; Supplementary Figure 4). Two months post-transplant, the average retention of implanted cells across the four experimental animals was 19% as measured by CFSE fluorescence (Figures 3C,D; Supplementary Figure 5). An average of 45% of the CFSE+ cells expressed the proliferation marker KI67, based on immunostaining, inferring that some of the implanted cells may be maintained through proliferation (Figure 3E). Troponin I, a cardiomyocyte marker, was co-expressed in an average of 35% of the CFSE + cells (Figures 3F,G; Supplementary Figures 6, 7) (28). We also conducted a connexin43 stain (Supplementary Figure 8). These results suggest that approximately one third of the transplanted cells differentiated towards a cardiomyocyte lineage as a consequence of the local environmental factors. Approximately half of CFSE+ cells were ISL1+ positive at the time of sacrifice (Figure 3H). *FOXA2*, a stem cell marker co-expressed on the ISL1+ cells at the time of cell injection, was elevated 48-fold in the cell-treated repair zone as identified using RT-qPCR (Figure 3I). To assess the potential mechanisms behind the retention and proliferative capacity of the neonatal ovine ISL1+ stem cells, an analysis of pro-survival pathways was conducted. Transcripts associated with AKT signaling were elevated in the region of stem cell transplantation when compared against both the non-infarct region and infarct-only controls (Figure 3J). This was validated by the elevated total pAKT protein level, the active pro-regenerative form of AKT, and a higher pAKT to AKT ratio (Figures 3L,M). AKT can induce cardiac regeneration via activation of YAP1, a critical factor in cardiac repair (29, 30). YAP1 can also be induced by the pro-regenerative protein AGRN (31). Both *YAP1* and *AGRN* transcripts were elevated in the cell-treated repair zone (Figures 3N,O). *NOTCH1, NOTCH2,* and *SOD2* transcripts were induced following transplantation (Figures 3P,Q) and a positive trend towards higher levels of cytoprotective transcripts *TNFAIP3* and *BCL2* was identified (Supplementary Figure 9). Collectively these genes have well-established roles in mitigating oxidative stress, and preventing apoptosis (32–34). Furthermore, *BAX*, an apoptosis marker, was not significantly altered when compared against the infarct-only control (Figure 3Q) or against the non-infarct zone (Supplementary Table 11).

### 3.4 Paracrine effects and endogenous cell recruitment within the cardiovascular repair zone

**Figure 4:**
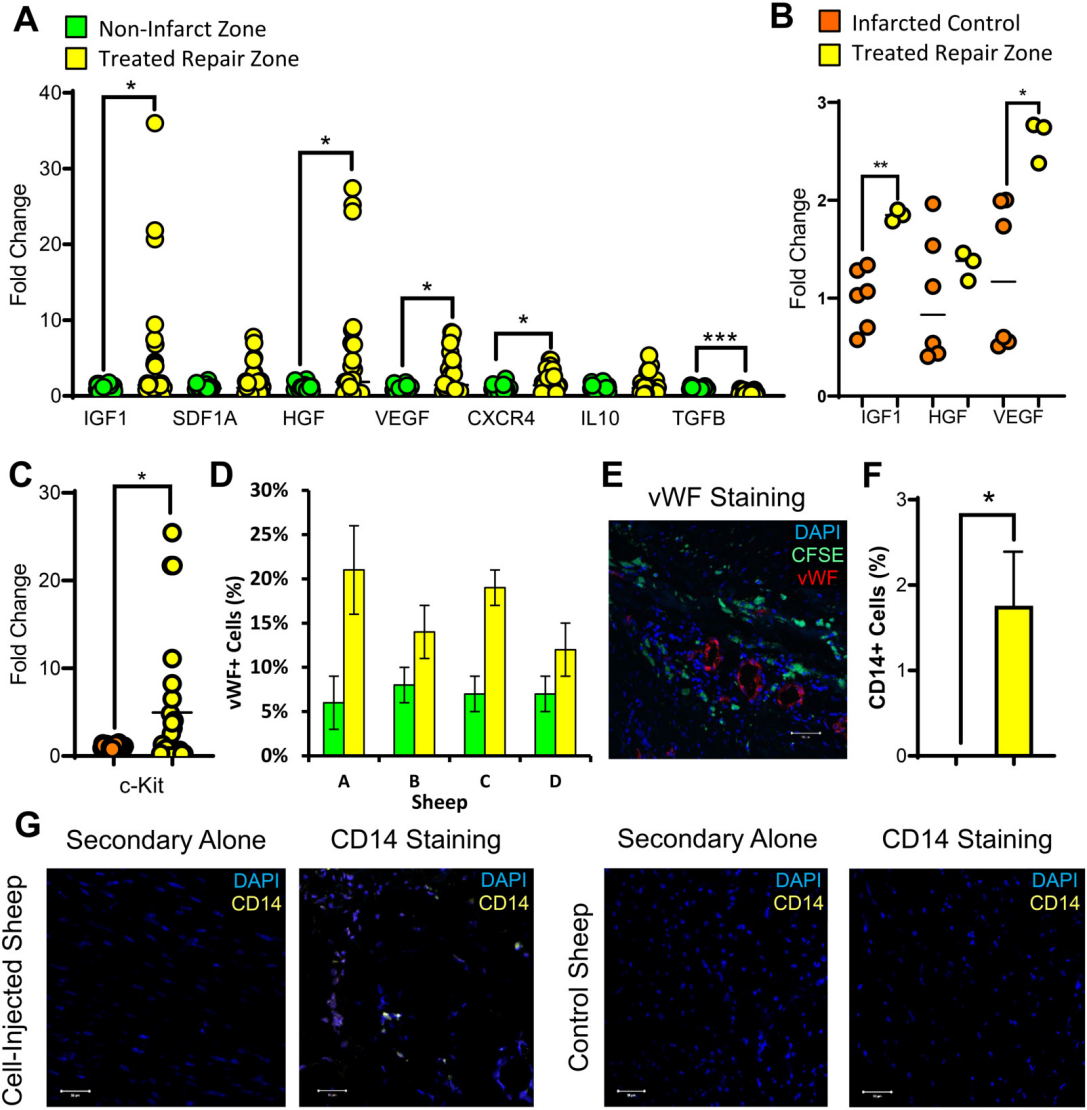
Host cell recruitment and vascularization markers are induced in the infarcted region of the heart following stem cell transplantation. Two months following ISL1+ MESP1+ FOXA2+ stem cell transplantation, cardiac tissue was obtained from the stem cell treated repair zone (yellow) and non-infarcted region of the heart (green). RT-qPCR was used to measure paracrine-related gene expression in the repair zone (yellow) compared against the non-infarct zone (green) **(A)** and against the infarct-only control (orange) **(B)**. In the repair zone of stem cell-treated (yellow) and control ewes (orange), the recruitment of CKIT-expressing cells was assessed using RT-qPCR **(C)**. Vascularization was assessed by the quantification of VWF-expressing cells within each stem cell-transplanted ewe **(D)** and visualized by the presence of VWF-expressing endothelial cells in the infarcted regions of the heart (**E:** DAPI, blue; CFSE, green; VWF, red). CD14, a common macrophage marker, was assessed via IHC **(F,G)** in the repair zone of stem cell-treated (yellow) and control ewes. The Shapiro–Wilk test or d'Agostine Pearson omnibus normality test were used to determine if data was normally distributed. To compare the means of normally distributed data, a Student's *t*-test was used while a Mann–Whitney *U* test was used for non-normally distributed data. Data is presented as the mean ± SEM, *n* = 1–4 ewes per group, each measured in triplicate. *p < 0.05, **p < 0.01; ***p < 0.001, ****p < 0.0001.

The efficacy of a stem cell therapeutic is partially determined by its capacity to induce endogenous tissue regeneration via paracrine signaling. Analysis of the cell-treated repair zone showed significant modulation of numerous paracrine markers (Figures 4A,B). Transcripts encoding growth factors and supporting angiogenesis, including *IGF1, HGF, VEGF,* and *CXCR4* were all significantly elevated. Meanwhile, *TGFβ*, which induces fibrosis, was significantly downregulated. Endogenous recruitment was further assessed by quantifying CKIT+ cells. CKIT+ cells were elevated in the cell-treated repair zone (Figure 4C). Revascularization was assessed by quantifying VWF+ cells which were elevated across all cell-treated sheep (Figures 4D,E; Supplementary Figure 10). We evaluated CD14 expression, a marker of infiltrating immune cells which play an important role in cardiac repair (35). Our results demonstrated significant CD14+ cell recruitment in cell-treated sheep (Figures 4F,G; Supplementary Figures 11–14).

### 3.5 Signaling pathways activated in the cardiac repair zone of the cell-treated heart

**Figure 5:**
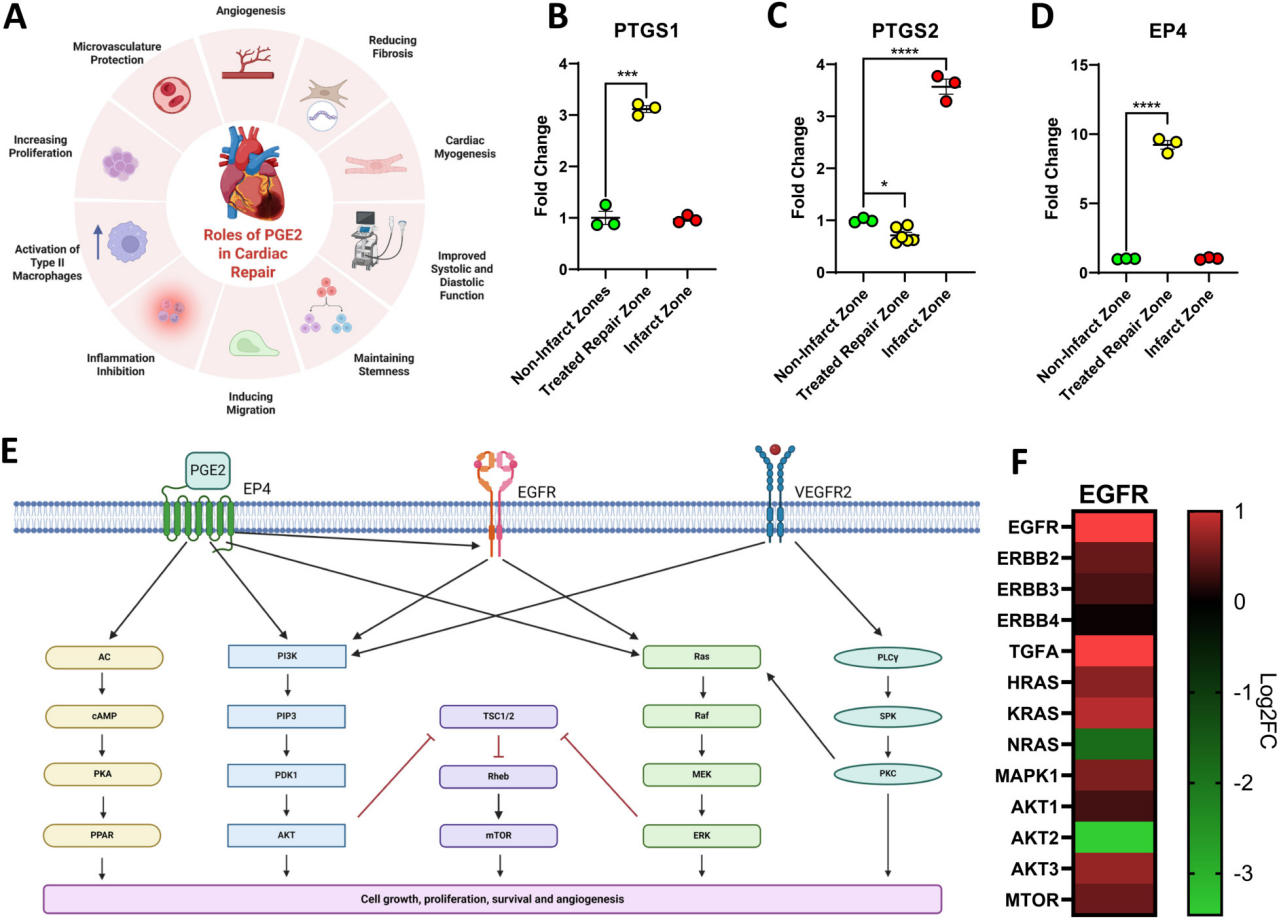
Signaling pathways activated in the cardiac repair zone of the cell-treated heart. Prostaglandin E2 (PGE2) has been previously documented to contribute to cardiac repair via numerous mechanisms **(A)**. Figure created with BioRender.com. Assessment of Prostaglandin synthases demonstrated that synthase 1 (PTGS1) showed a significant elevation in the cell-treated region (yellow) when compared to the non-infarcted region (green) while synthase 2 (PTGS2) was only elevated in the infarct region (red) **(B,C)**. EP4, a PGE2 receptor that can directly induce regeneration and repair, was only elevated in the cell-treated repair zone (yellow) **(D)**. Differentially expressed genes identified as significantly expressed in the cell-treated zone using RNAseq were uploaded to NCBI DAVID and OmicsNet. The EGFR signaling pathway was predicted to be involved in cardiac repair within the cell-treated zone of three ISL1+ stem cell-treated sheep **(E)**. The pathway was generated using Biorender.com. Gene expression of key EGFR signaling components is visualized via heatmap **(F)**. Data is presented as the mean ± SEM, *n* = 1 representative ewe, measured in triplicate shown. Trend repeatable in *n* = 3 tested ewes. *p < 0.05, **p < 0.01; ***p < 0.001, ****p < 0.0001.

Due to the well-documented roles of PGE2 in cardiac repair (Figure 5A) (36), the expression of various components of the PGE2 signaling pathway were examined. Prostaglandin Synthase 1 (*PTGS1*) was elevated in the repair zone however Prostaglandin Synthase 2 (*PTGS2*) was not (Figures 5B,C). Prostaglandin Receptor 4 (*EP4*), a key receptor that enhances cardiac repair, promotes angiogenesis, and mitigates fibrosis, was elevated in the cardiac repair zone (Figure 5D) (37, 38). These findings suggest that there is an enriched capacity to produce and respond to PGE2 in the cell-treated repair zone which may contribute to elevated levels of KI67+, VWF+ and CD14+ cells and to the downregulation of *TGFβ*. To further elucidate the mechanisms contributing to the regenerative effects identified in the cell-treated region of the infarcted heart, a transcriptomic analysis on the tissue was conducted. All significant differentially expressed genes in the cell-treated zone relative to the non-infarcted zone were uploaded to NCBI DAVID and OmicsNet using the InnateDB database (39, 40). REACTOME analysis predicted modulation of the EGFR signaling pathway. The coordination of this pathway with EP4 and VEGFR signaling pathways is shown in Figure 5E. The predicated pathway activation identified by transcriptomic analysis coincides with the regulation of AKT, angiogenesis, and PGE2 (Figures 3J–M, 4A–E, 5A–D). The subsequent predicted activation of cAMP, mTOR, and MAPK signaling may facilitate cell survival and proliferation.

### 3.6 ISL1+ stem cells restore cardiac function to pre-infarct levels two months post-transplant

**Figure 6:**
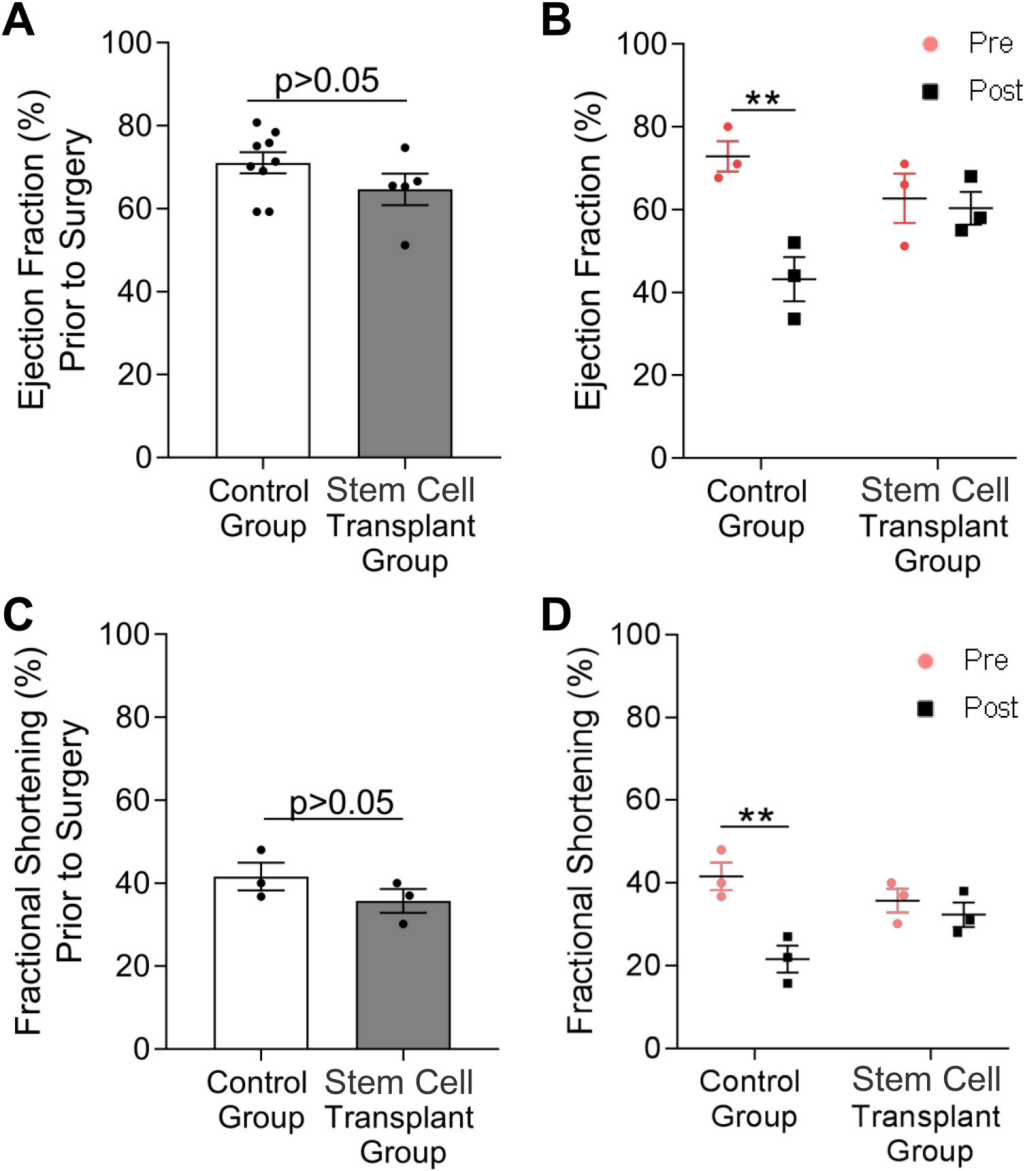
Cardiac function recovers in ewes treated with neonatal ISL1+ MESP1+ FOXA2+ stem cells. Echocardiography was performed to measure ejection fraction **(A,B)** and fractional shortening **(C,D)** before LAD ligation and at two months post stem cell transplant. Echocardiography revealed no difference in ejection fraction and fractional shortening between control and stem cell-treated ewes prior to surgery **(A,C)**. Meanwhile, a significant decrease in both ejection fraction and fractional shortening was measured in control ewes at three months after surgery; however, both measures were nearly unchanged in stem cell-treated ewes **(B,D)**. A two-way ANOVA was performed to measure the effect of stem cell transplantation on cardiac functional measures within and across groups. Data is presented as the mean ± SEM, *n* = 3−4 ewes per group, each measured in triplicate **p < 0.01.

To identify the functional impact of implanted neonatal ovine *ISL1+ MESP1+ FOXA2+* stem cells on cardiac regeneration post myocardial-infarction, ejection fraction (EF) and fractional shortening (FS) were assessed in the control and cell-treated sheep via echocardiogram. Specifically, sheep ≤1 year of age were separated into two groups of three, an infarct-only control group and an infarct with cell-treatment group. Sheep in both groups received echocardiograms prior to infarction via LAD ligation and at the time of sacrifice three months post-infarct. Both ejection fraction and fractional shortening were not significantly different between the control and treated groups prior to LAD ligation (Figures 6A,C). The infarct-only control group showed a significant reduction in both measures three months post infarct (Figures 6B,D). Ejection fraction was reduced from 71% ± 2.6 to 39% ± 2.8 and FS was reduced from 40% ± 2.1 to 20% ± 1.8. In contrast, the cell-treated group showed no significant difference in ejection fraction or fractional shortening when compared to pre-infarct levels (Figures 6B,D). Ejection fraction in cell-treated sheep prior to LAD was 65% ± 3.8 and two months post ISL1+ stem cell transplant, the ejection fraction was 60% ± 3.4. Fractional shortening of the treated sheep was initially 36% ± 2.6 prior to infarction and 32% ± 2.6 at the time of sacrifice. Histological examination of the cell-treated tissue showed no evidence of teratoma or tumor formation. In this model, the neonatal ovine ISL1+ stem cells demonstrated the ability to rescue cardiac function post-infarction.

## 4 Discussion

In this report, we characterize the transcriptome and functional potential of neonatal *ISL1+ MESP1+ FOXA2+* stem cells and demonstrate that these early-stage stem cells can restore cardiac function to pre-infarct levels in an ovine model of MI. Neonatally-derived stem cells divide rapidly and can be expanded as clonal populations from the cardiovascular tissue of human neonates or newborn sheep for use in preclinical efficacy studies (14, 15). This report is the first, to our knowledge, to examine the functional efficacy and mechanistic basis of repair following administration of neonatal *ISL1+ MESP1+ FOXA2+* stem cell clones in a sheep model of MI.

Neonatal cardiovascular tissue is enriched with ISL1+ cells during the pro-regenerative period shortly after birth (41, 42). ISL1+ progenitors contribute to the majority of the cells of the heart and previous studies showed that they promote cardiac repair in small animal models (43, 44). Our transcriptomic analysis of neonatal ISL1+ stem cells has identified several unique characteristics, including similarities in expression patterning to iPSCs at early stages of differentiation (7). Human neonatal *ISL1+ MESP1+ FOXA2*+ stem cells express multiple markers associated with the epiblast, the primitive streak and mesodermal progenitors including *SALL4* and *TFAP2C*, yet they did not develop into tumors when administered for cell-based repair *in vivo* (7, 16). Transcriptomic analysis suggested that cardiovascular tissue obtained from neonates represents a novel and alternative source of early-stage stem cells for cardiovascular repair.

There are several major challenges associated with stem cell-based therapies including difficulty with achieving consistent, lasting functional improvement, limited cell retention, and safety. Restoration of cardiac function and significant retention has recently been shown in large animals using fully differentiated cardiomyocytes derived from iPSCs and ESCs, although transient arrhythmias represent a challenge in these models. Two notable studies in non-human primates have demonstrated significant functional improvement utilizing between 750 million and 1 billion hESC-derived fully differentiated cardiomyocytes (2, 3). Autologous iPSC-derived cardiomyocytes have been used in non-human primate studies without the need for immunosuppression, demonstrating both successful engraftment and *in vivo* maturation without teratoma. Allogeneic transplants, however, were rejected within 8 weeks (5). Early-stage progenitors have also been used in large animal models with promising results. Intravenous injection of undifferentiated multilineage-differentiating stress enduring (MUSE) cells in a mini pig model improved cardiac function without immunosuppression. In this model, cells exhibit selective cell homing to the border region post injury (45). Notably, MUSE cells are distinct from the ISL1+ stem cells reported in our study based on transcriptomic expression patterns and origin (46). Preclinical studies using early-stage cells from other sources, specifically hESC-derived SSEA1+ multipotent cardiac progenitor cells, have been shown to reduce scar tissue by 20%, a finding of particular interest to us as these cells also express *MESP1* and *ISL1* (47). This population appears to share similarities with the early-stage stem cells described here despite the different cell source. The same group recently conducted a Phase I clinical trial wherein patients received a median dose of 8.2 million *ISL1+ SSEA1+* hESC-derived cardiac progenitor cells which improved cardiac function in a small number of patients, none of whom experienced any arrhythmias during the duration of the study (10).

The regenerative zone of the infarcted heart provided us with significant insight into the mechanistic basis behind cardiovascular repair in our model. The pro-regenerative AKT-signaling pathway, which is involved in cell proliferation, survival, migration and cardioprotection was induced (48). Furthermore, *AGRN* and *YAP1* transcripts were elevated in the cardiovascular repair zone. YAP1 alone is sufficient to induce cardiac regeneration in small animal models (29). Paracrine signaling molecules which influence endogenous recruitment of cells were elevated, including IGF1 and HGF whose overexpression in MSCs was previously shown to improve vascularization and reduce inflammation in a porcine MI model (49). *IGF1, HGF, VEGF* and *CXCR4* transcripts are all associated with angiogenesis and contribute to cardiovascular repair in our model (50, 51).

Stimulation of angiogenesis can also be mediated by PGE2 (37). The well-documented benefits of PGE2 in cardiovascular repair also include activation of proliferation in cardiomyocytes, inhibition of fibrosis and modulation of the immune response (36, 52, 53). The elevated expression of *PTGS1* and *EP4* in the cardiac repair zone noted in our study suggests that PGE2 could be contributing to regeneration in the sheep model of MI and stem cell-based repair. PGE2 has previously been shown to prevent the rejection of implanted allogeneic MSCs (54), suggesting that *ISL1+ MESP1+ FOXA2*+ stem cell retention in the absence of immunosuppressive drugs may partially be attributed to PGE2 signaling in the repair zone. PGE2 also downregulates *TGFβ* production in cardiomyocytes, protecting against heart failure and reducing the effects of fibrosis (55). Furthermore, EP4 overexpression alone has been shown to improve cardiac function (56). The inflammatory microenvironment induced following injury is a crucial component of successful cardiac repair. Much of this is determined by the local recruitment of specific immune cell populations (57). PGE2 has been reported to play a role in the regulation of macrophage recruitment in the ischemic heart which aligns with the elevated number of CD14+ cells recruited into the ISL1+ cell-treated sheep hearts reported here (58). This is significant as PGE2 can convert M1 pro-inflammatory macrophages to M2 regenerative macrophages (59). Additionally, in late-stage ischemic injuries, similar to our study, resident macrophages proliferate and encourage repair which could also contribute to our reported findings (60). While we do not have causal proof for the contribution of PGE2 to the beneficial effects reported in our study, taken together it is likely that PGE2 expression contributes to the recruitment, retention, and functional outcomes noted in this model.

While recent reports of cardiac repair using embryonic stem cells, induced pluripotent stem cells and MUSE cells have been encouraging, these cell types pose challenges associated with rapid isolation, expansion and purity. In this report, we used a novel approach for cell-based cardiac repair wherein we tapped a unique resource known for enhanced regenerative ability which allowed us to rapidly isolate and clonally select stem cells with regenerative capacity which can be used for immediate transplantation or stored as a pure population for later use as an off-the-shelf therapeutic. The neonatal regenerative window is a well-documented transient timeframe during which regenerative stem cells with significant advantages exist in humans. We demonstrate here that *ISL1+ MESP1+ FOXA2+* stem cells isolated during this timeframe have the ability to differentiate into all cells of the cardiovascular lineage, they are retained without immunosuppression when administered for cell-based cardiac repair and they restore cardiac function in large animals which is a challenging step when translating preclinical studies towards potential applications in humans. The transcriptomic analysis provided facilitates reproducibility of this data at other centers. The limitations to this work include the small number of animals used in this pilot, proof-of-concept study. Females were chosen since female patients exhibit clinically worse cardiac outcomes post cardiac insult when compared to male patients, allowing us to test the cells in a more challenging and clinically impactful model. Future work in additional animals of both sexes will be needed to further extend our knowledge about this novel, alternative source of early-stage stem cells with regenerative potential.

## Data Availability

The human transcriptomic datasets analyzed for this study can be found in the Gene Expression Omnibus database (GEO) GSE254267. Any additional information is available from the corresponding author upon reasonable request.
