# Supplementary material for: Neonatally-derived multipotent Islet-1+ Mesp1+FOXA2+ stem cell clones restore cardiac function in sheep
Source: Front Cardiovasc Med. 2026 Jan 5;12:1671367. doi: 10.3389/fcvm.2025.1671367 (PMC12812543; doi:10.3389/fcvm.2025.1671367)
Supplement: Supplementary file 1 [file Datasheet1.pdf]

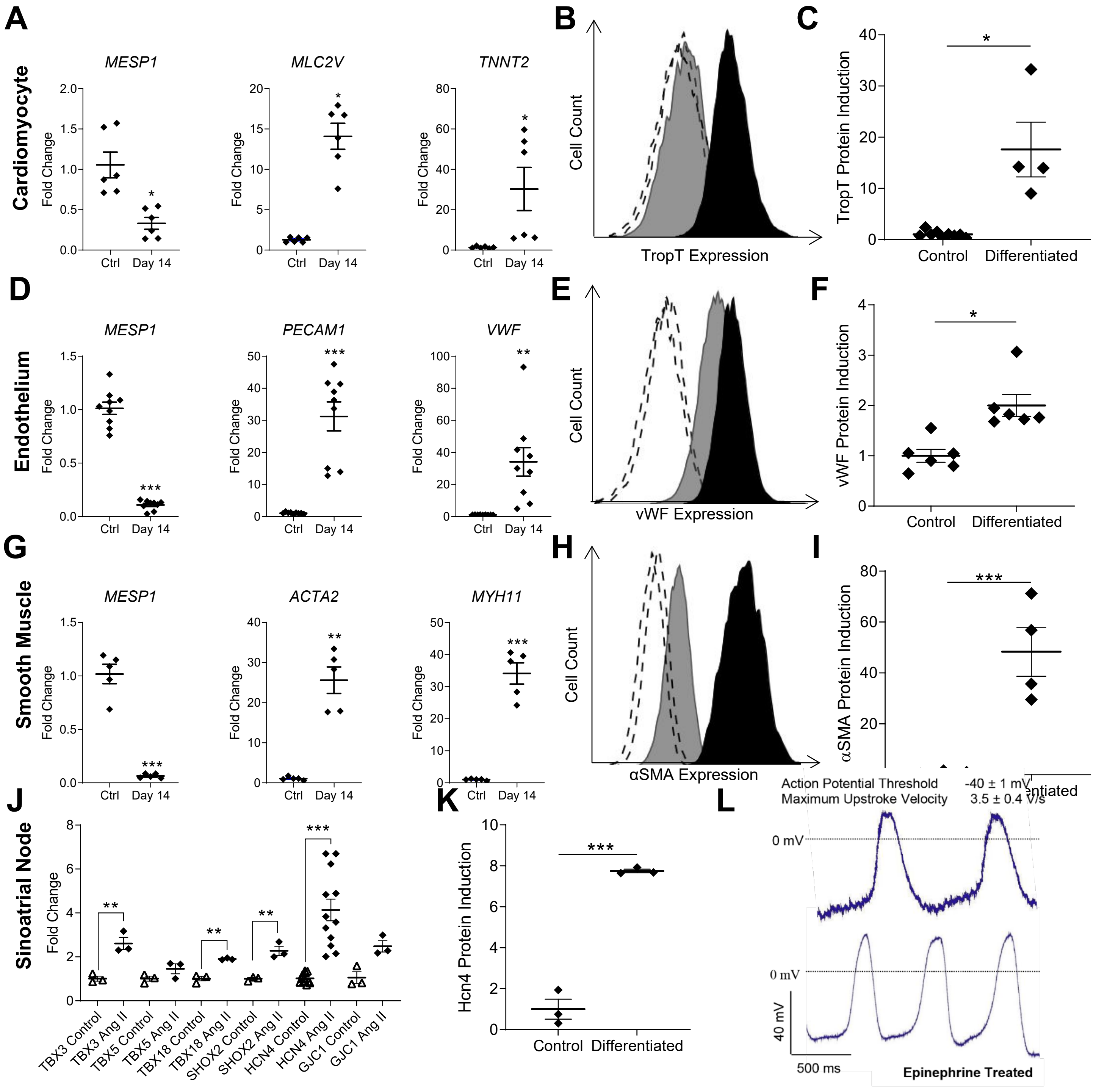

Supplementary Figure 1. Neonatal human stem cells differentiate into various cardiovascular lineages. Directed differentiation protocols were used to induce the development of cardiomyocytes (A-C), endothelium (D-F), smooth muscle (G-I), and sinoatrial nodal cells (J-L). After two weeks of differentiation, RT-qPCR was used to assess the expression of markers of the three major cardiovascular lineages (A, D, G), which was confirmed by flow cytometry (dash line, unstained; grey, control; black, differentiated; B, C, E, F, H, I). After 72 hours, RT-qPCR was used to assess the expression of markers of the sinoatrial node (J), which was confirmed by western blot (K) and electrophysiology experiments

(L). To determine normality of data distribution a Shapiro-Wilk test or d'Agostine Pearson omnibus normality test, when applicable, was used. To compare the means of protein and transcript expression, a Student's t-test was used to test normally distributed data while a Mann-Whitney U test or Wilcoxon signed rank test was used where appropriate, to test non-normally distributed data. Data is presented as the mean ± SEM, n=3-12 for all experiments. *$p<0.05$; **$p<0.01$; ***$p<0.001$

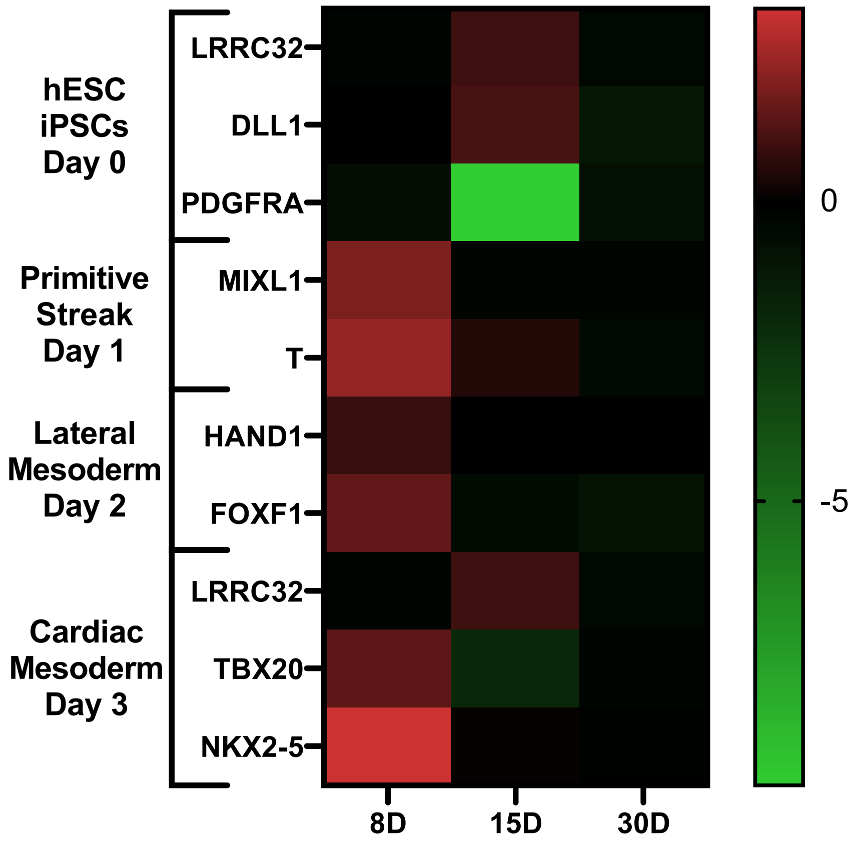

Supplementary Figure 2. The transcriptome of a clone isolated from 8 Day-old neonate aligns most closely with Day 1-2 of Differentiation

The clone isolated from an 8-day old neonate shows no expression of LRRC32/GARP, DLL1, or PDGFRA consistent with day 0 a study by Loh et al.. However, this clone possesses high expression of primitive streak markers MIXL1 and brachyury (T), and lateral mesoderm markers HAND1 and FOXF1. While the 8D clone does express cardiac mesodermal markers NKX2.5 and TBX20 these transcripts are not exclusive to day 3 and have been reported to be expressed as early as D1.5-D2 in Loh at al., 2016. Furthermore, another cardiac mesodermal marker, LRRC32/GARP has been used to define cardiac mesoderm at day 3 and it is not expressed in the 8D clone. Collectively this suggests that our 8-day clone most closely associates with day 1-2 of mesodermal differentiation towards a cardiac lineage.

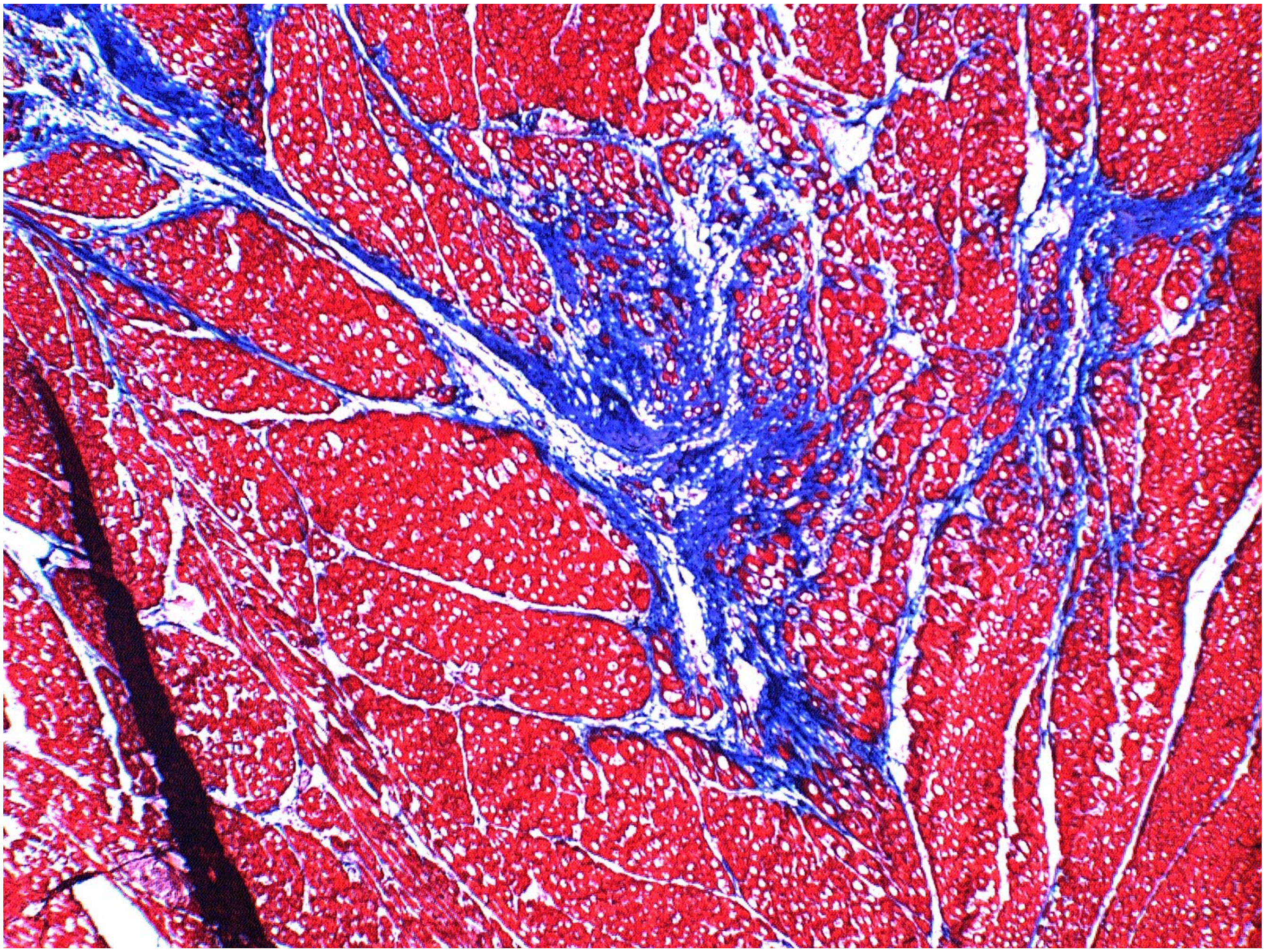

Supplementary Figure 3. Trichrome stained left ventricle area post infarct and stem cell transplantation.

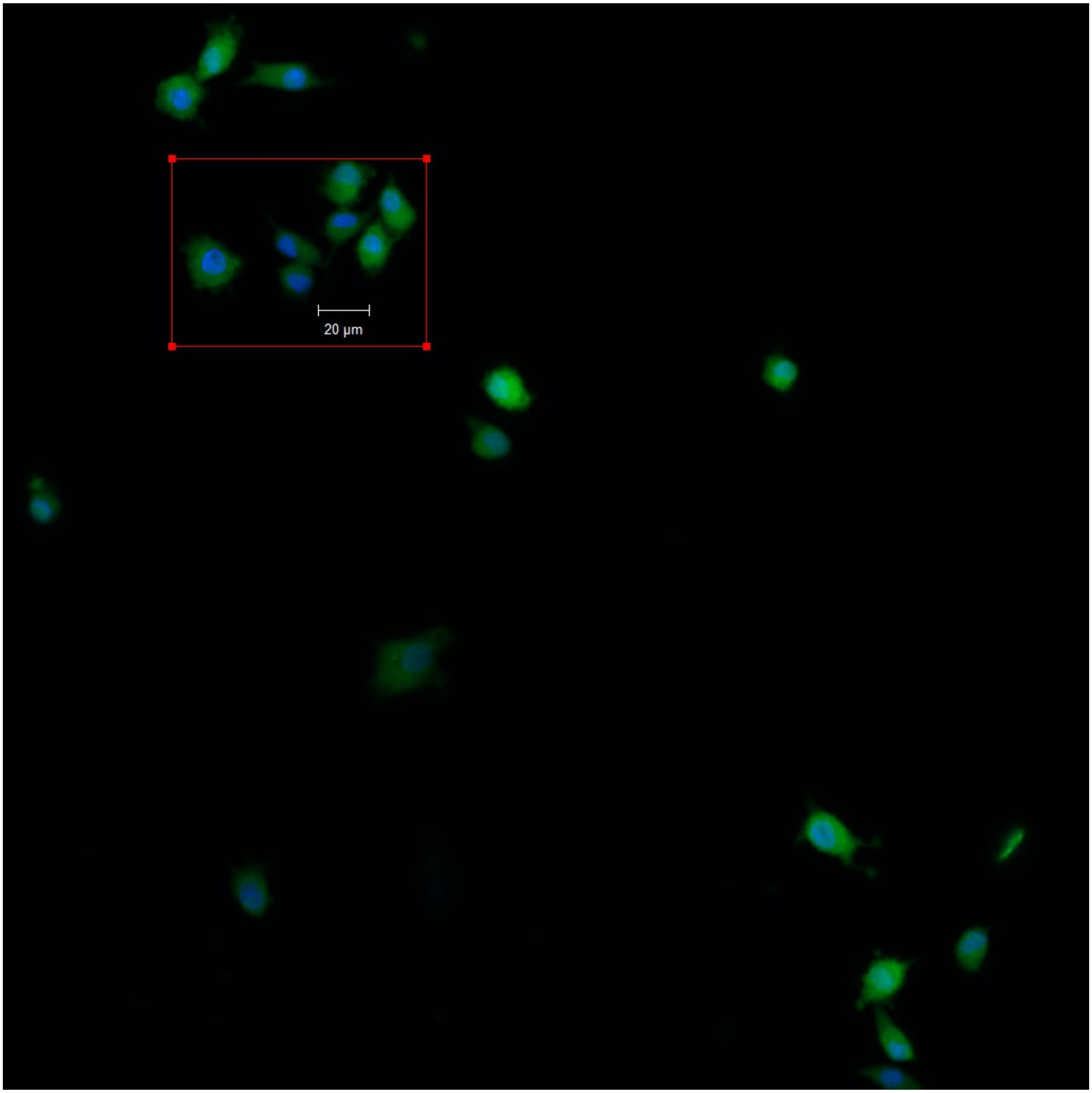

Supplementary Figure 4. High resolution version of Figure 3B

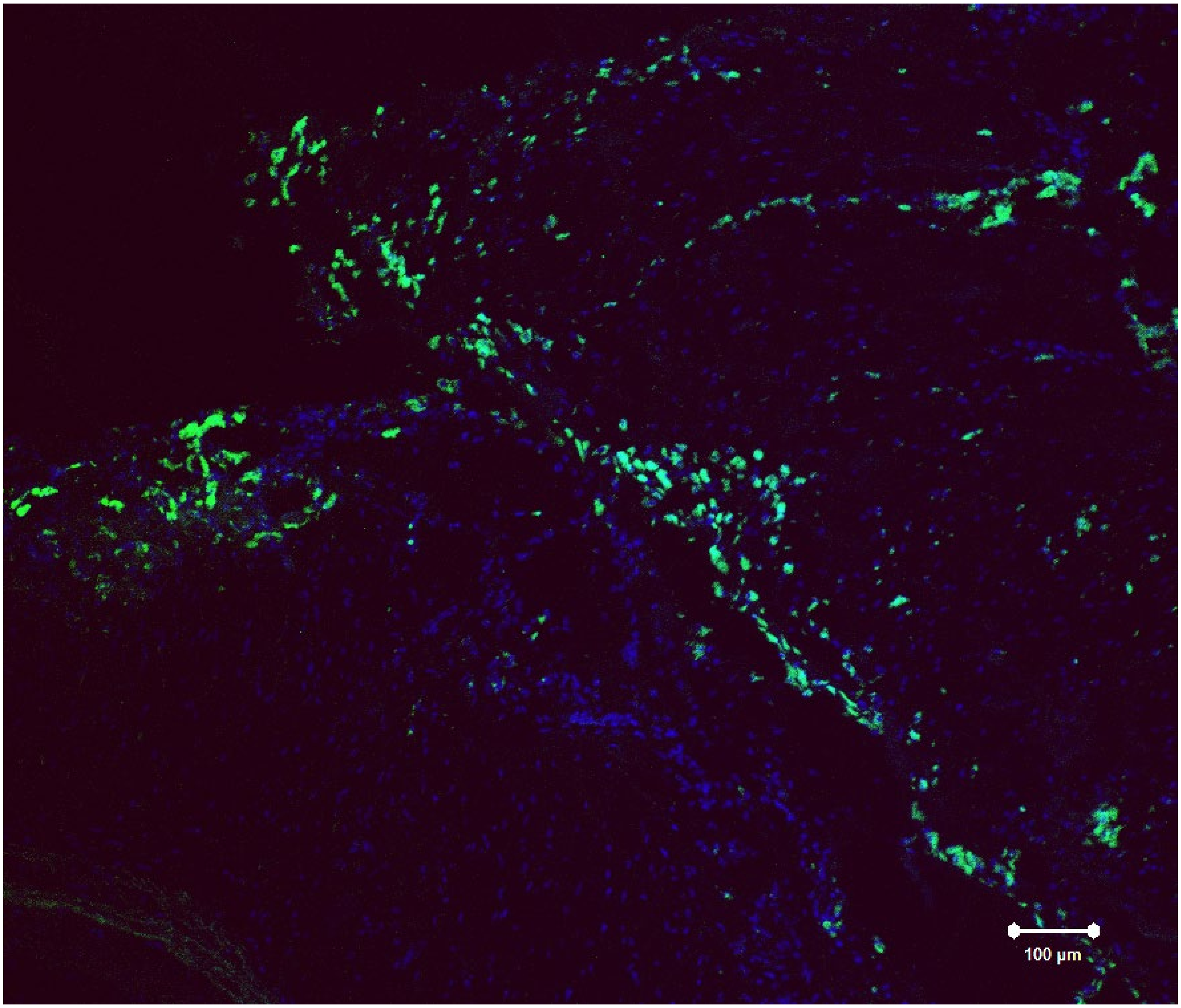

Supplementary Figure 5. High resolution version of Figure 3D

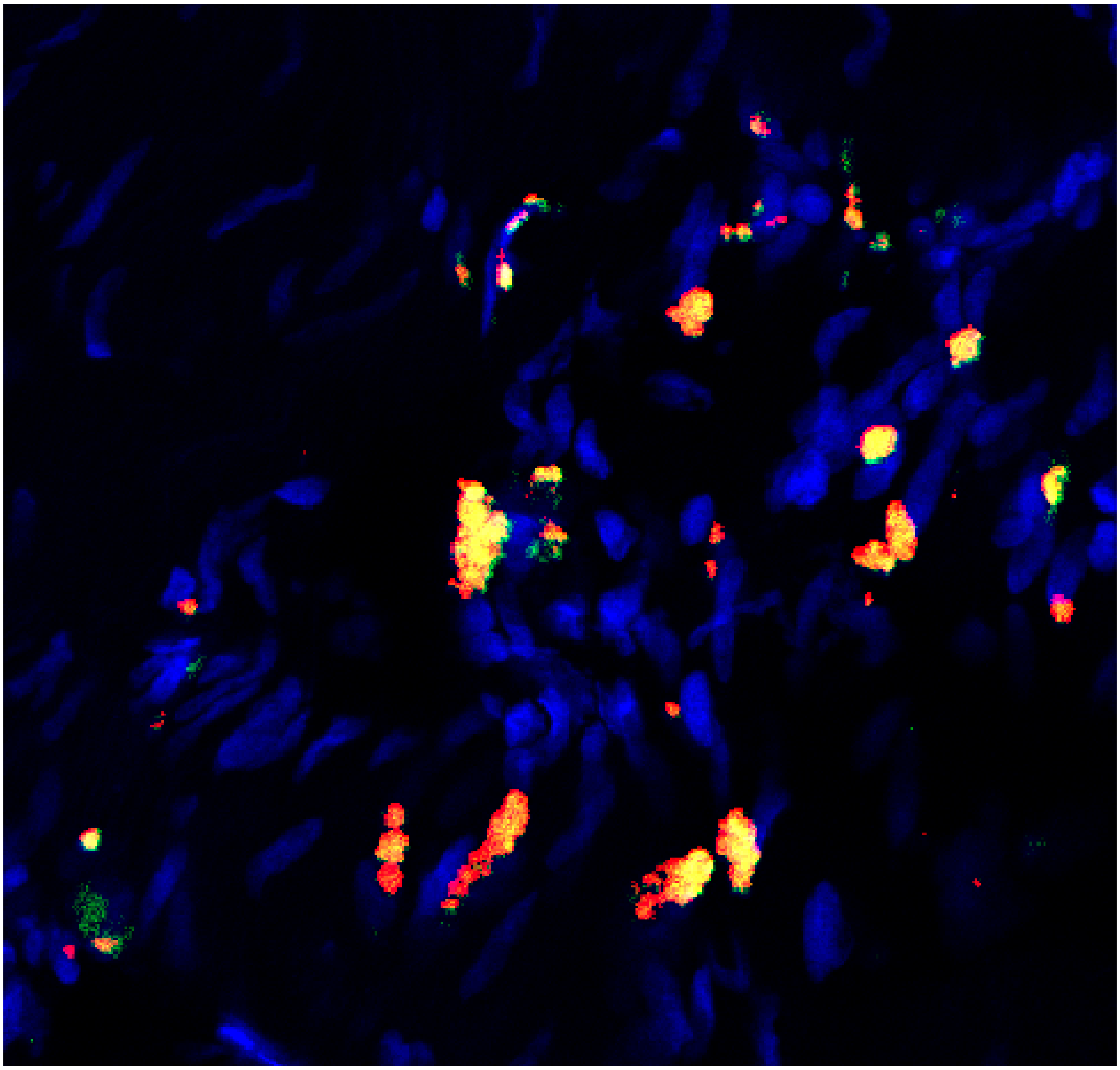

Supplementary Figure 6. High resolution version of Figure 3G

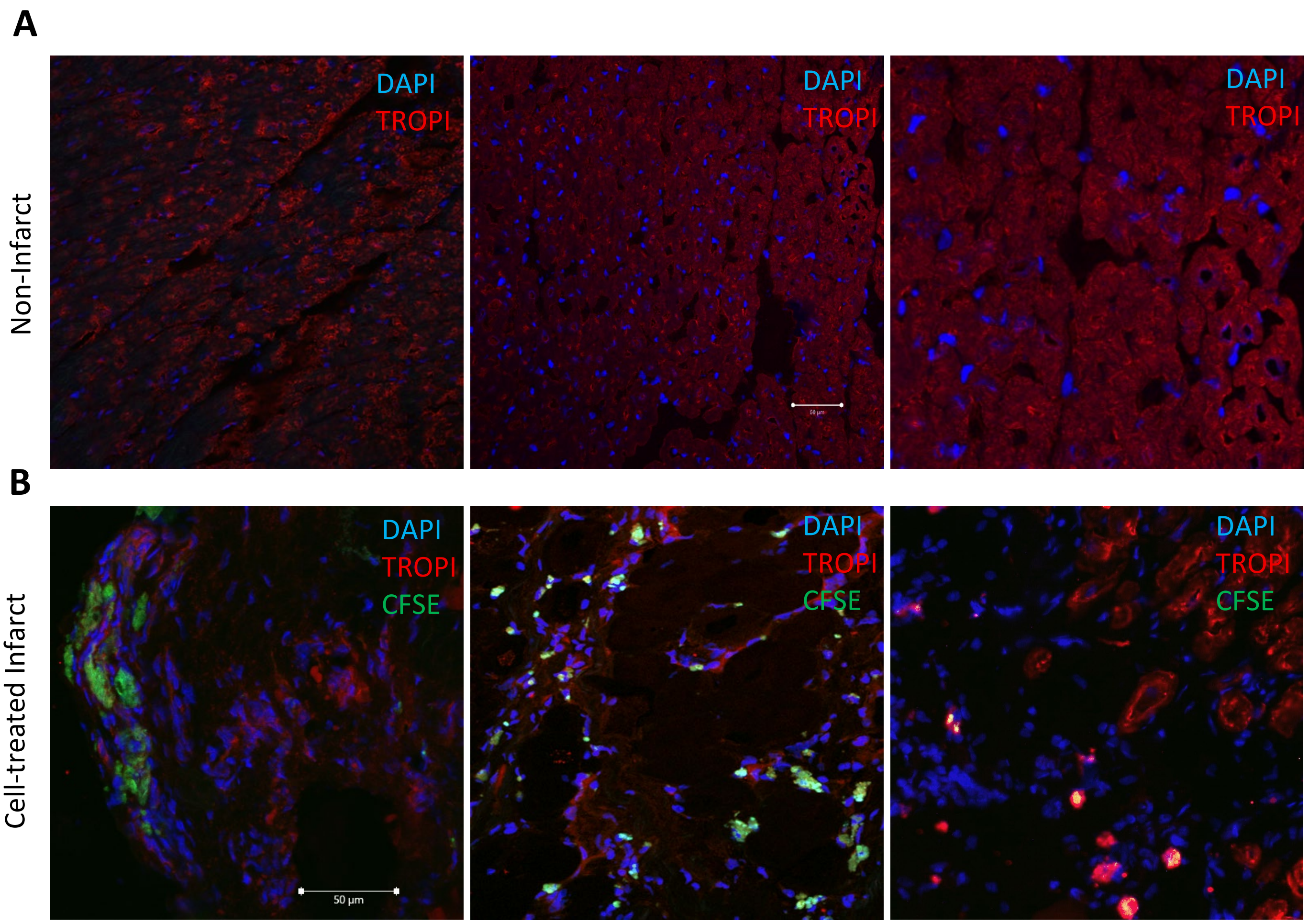

Supplementary Figure 7. Troponin I Stained Sections from both Non-Infarct and Infarcted, Cell-Treated Regions of the Heart

Troponin I staining (red) identifies cardiomyocytes in the non-infarct region where the last panel represents an enlarged image demonstrating specificity of Troponin I binding (A). Troponin I staining identifies host-derived cardiomyocytes (red) within the infarcted, cell-treated region of the heart in conjunction with stem cells tagged with CFSE (green) and select stem cells which demonstrate two color fluorescence suggesting differentiation of newly introduced stem cells into cardiomyocytes (B).

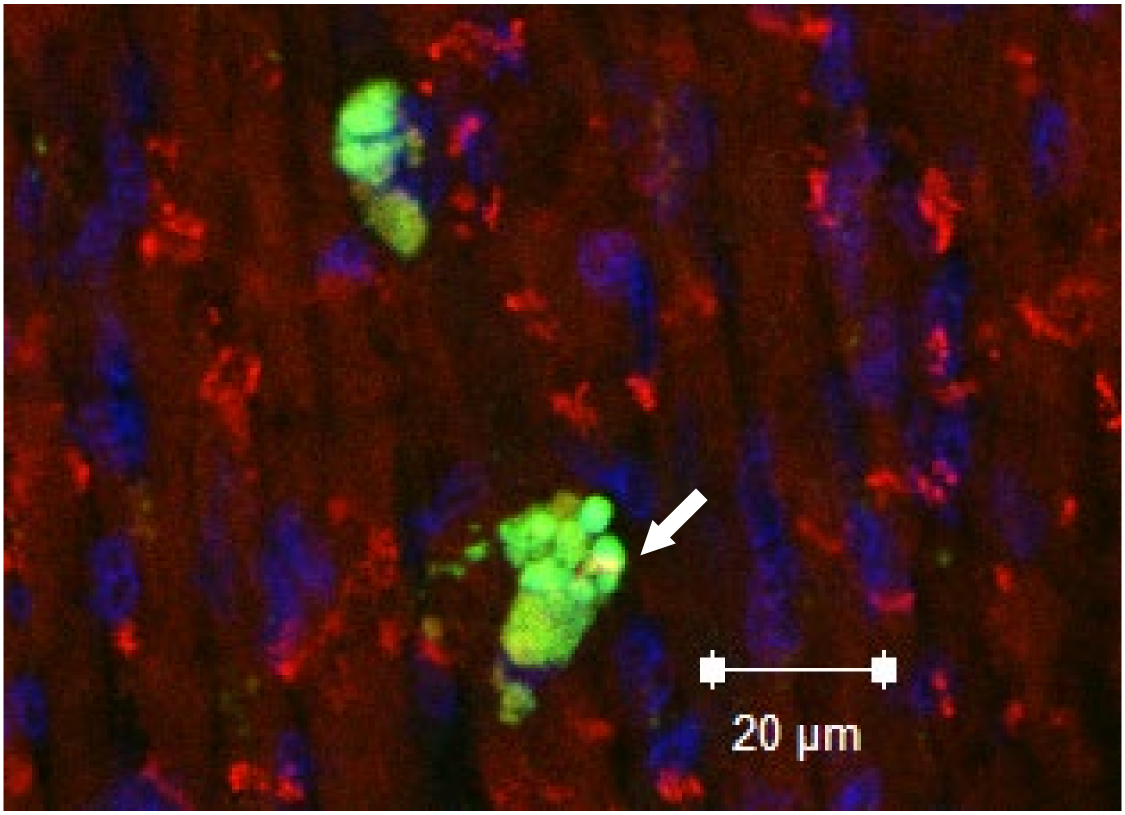

Supplementary Figure 8. Connexin 43 Stained Cells in the Cell-Treated Myocardium

Connexin 43 is a gap-junction protein whose presence is an indicator of intercellular communication. Connexin 43 is labeled red. CFSE+ stem cells are labeled green. Co-localization of introduced stem cells and connexin 43 appears as yellow.

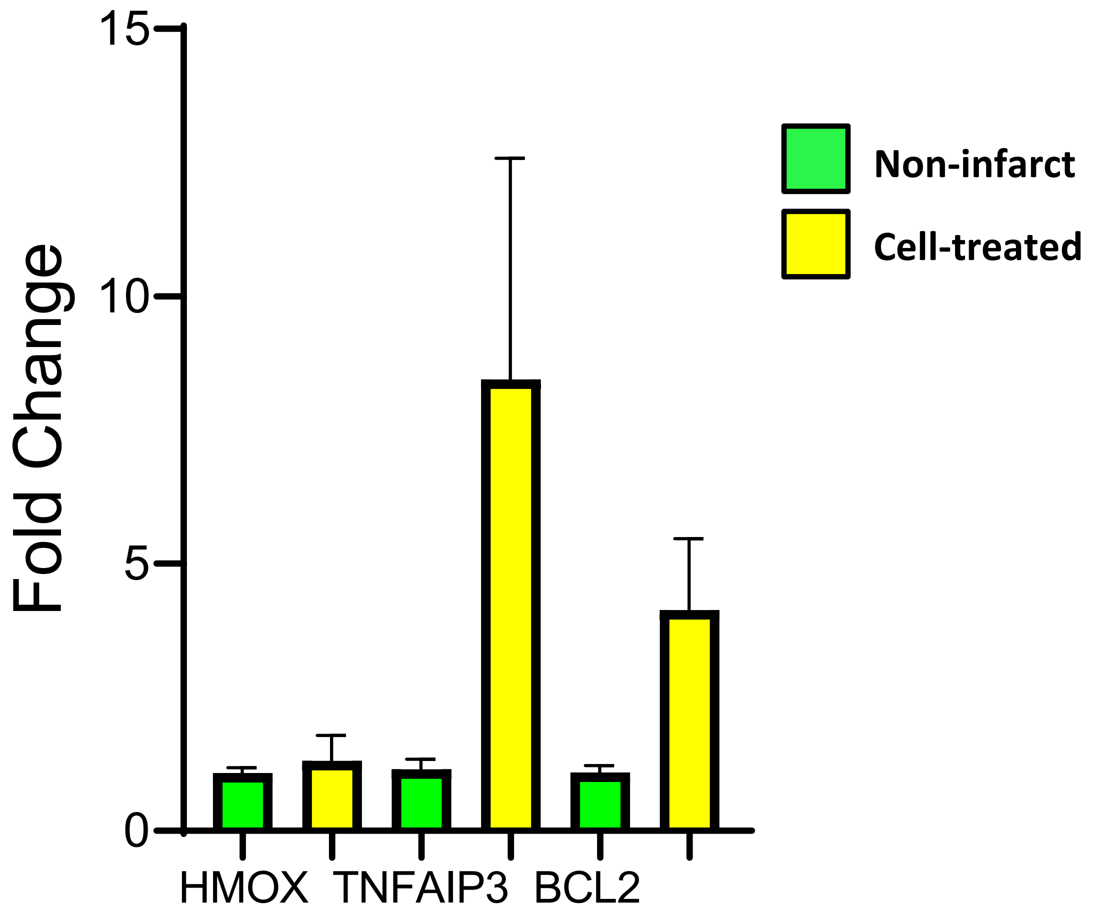

Supplementary Figure 9. Cardioprotective transcripts TNFAIP3 and BCL2 were elevated in the cardiovascular repair zone, however the trend was not statistically significant.

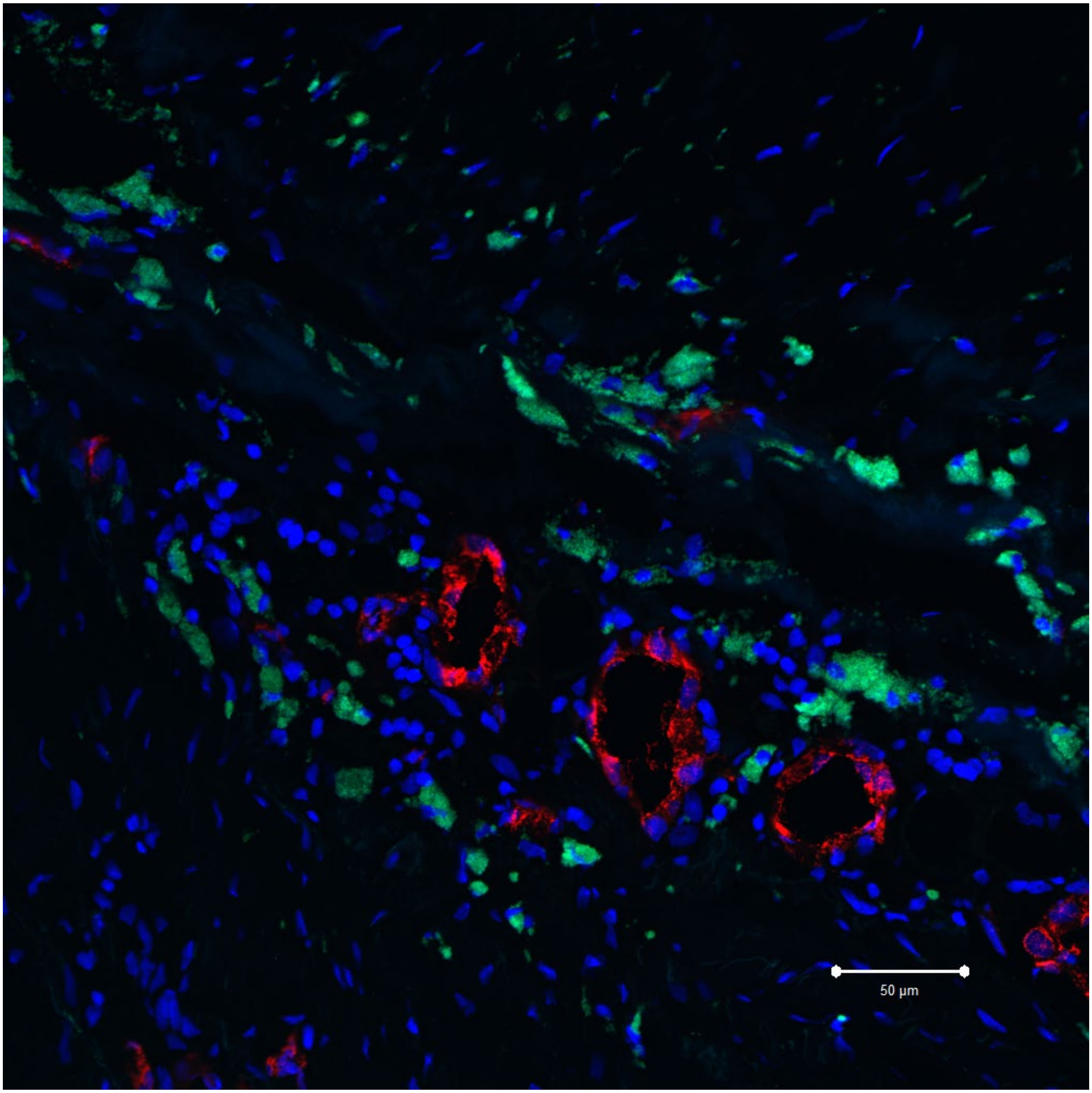

Supplementary Figure 10. High resolution version of Figure 4E

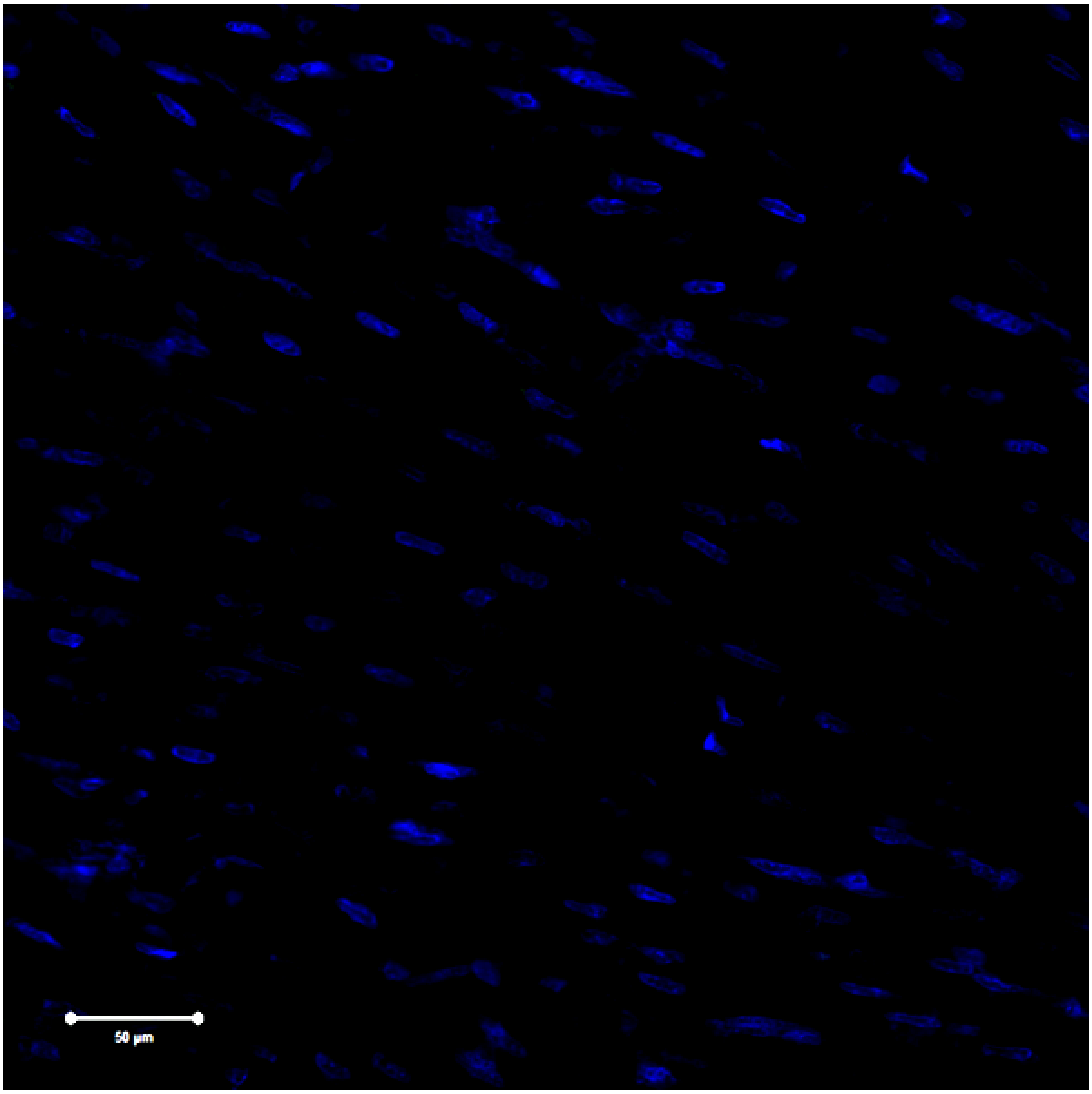

Supplementary Figure 11. High resolution version of Figure 4G (Cell Injected – Secondary Alone)

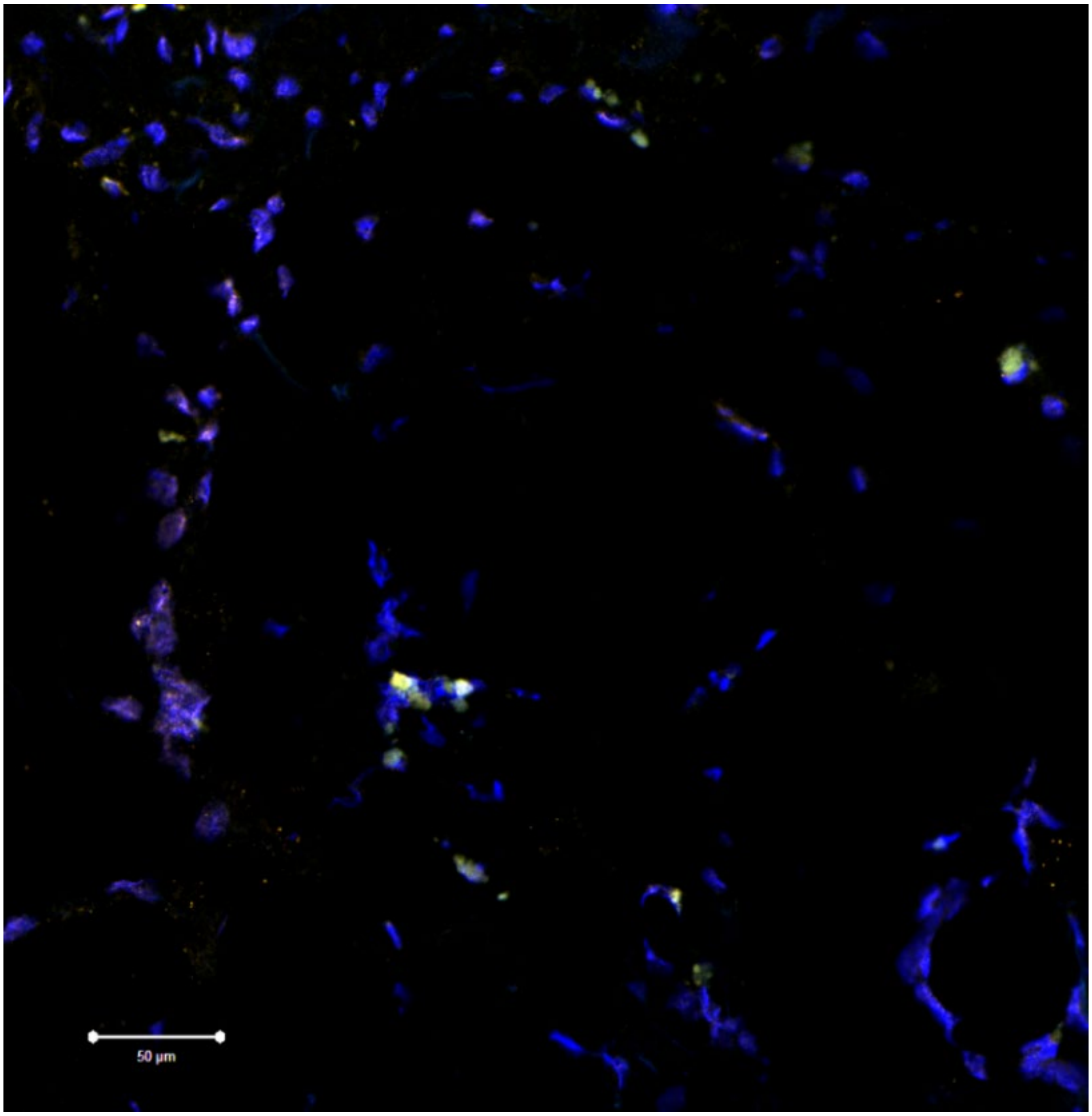

Supplementary Figure 12. High resolution version of Figure 4G (Cell Injected – CD14 Staining)

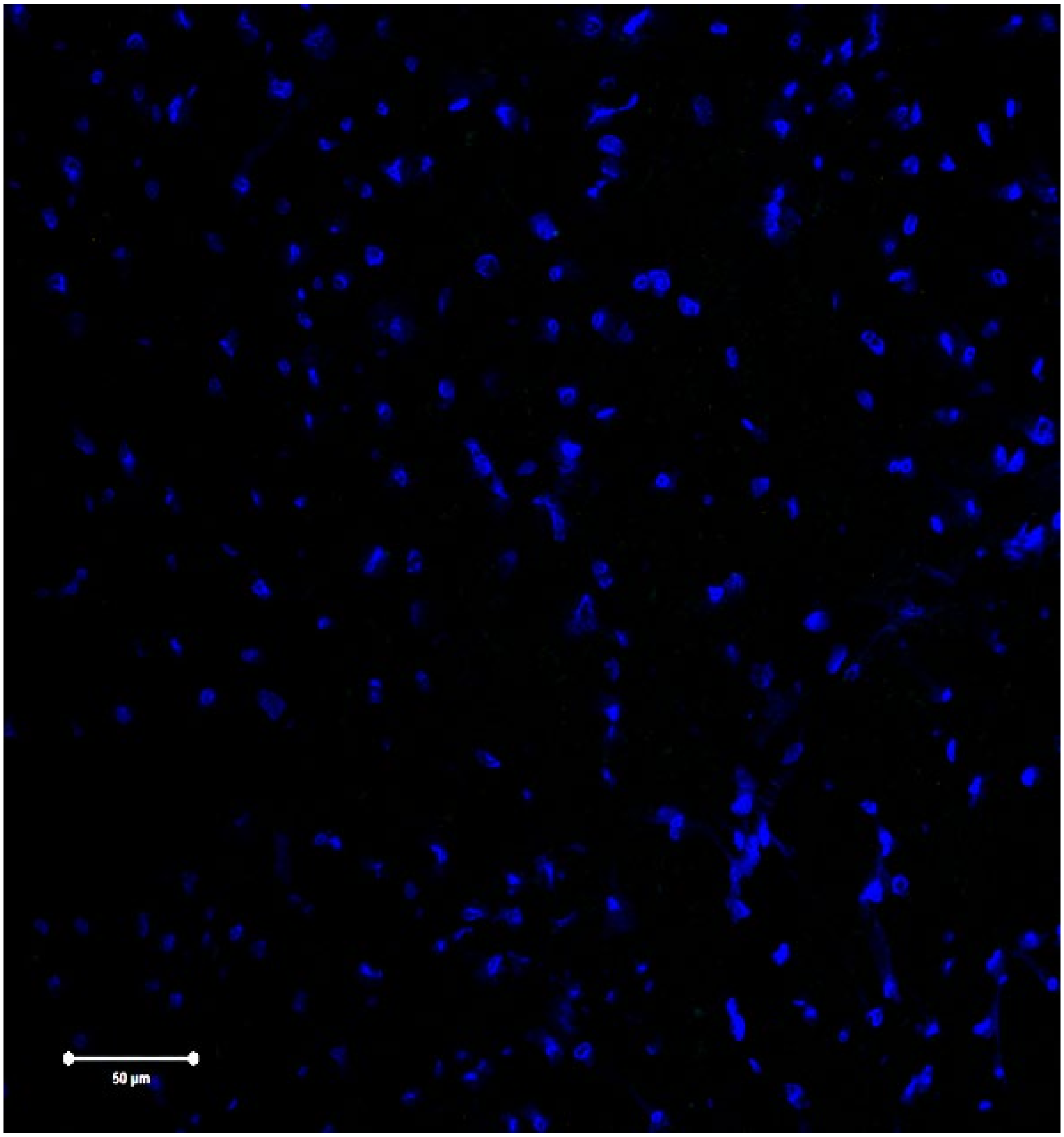

Supplementary Figure 13. High resolution version of Figure 4G (Control Sheep – Secondary Alone)

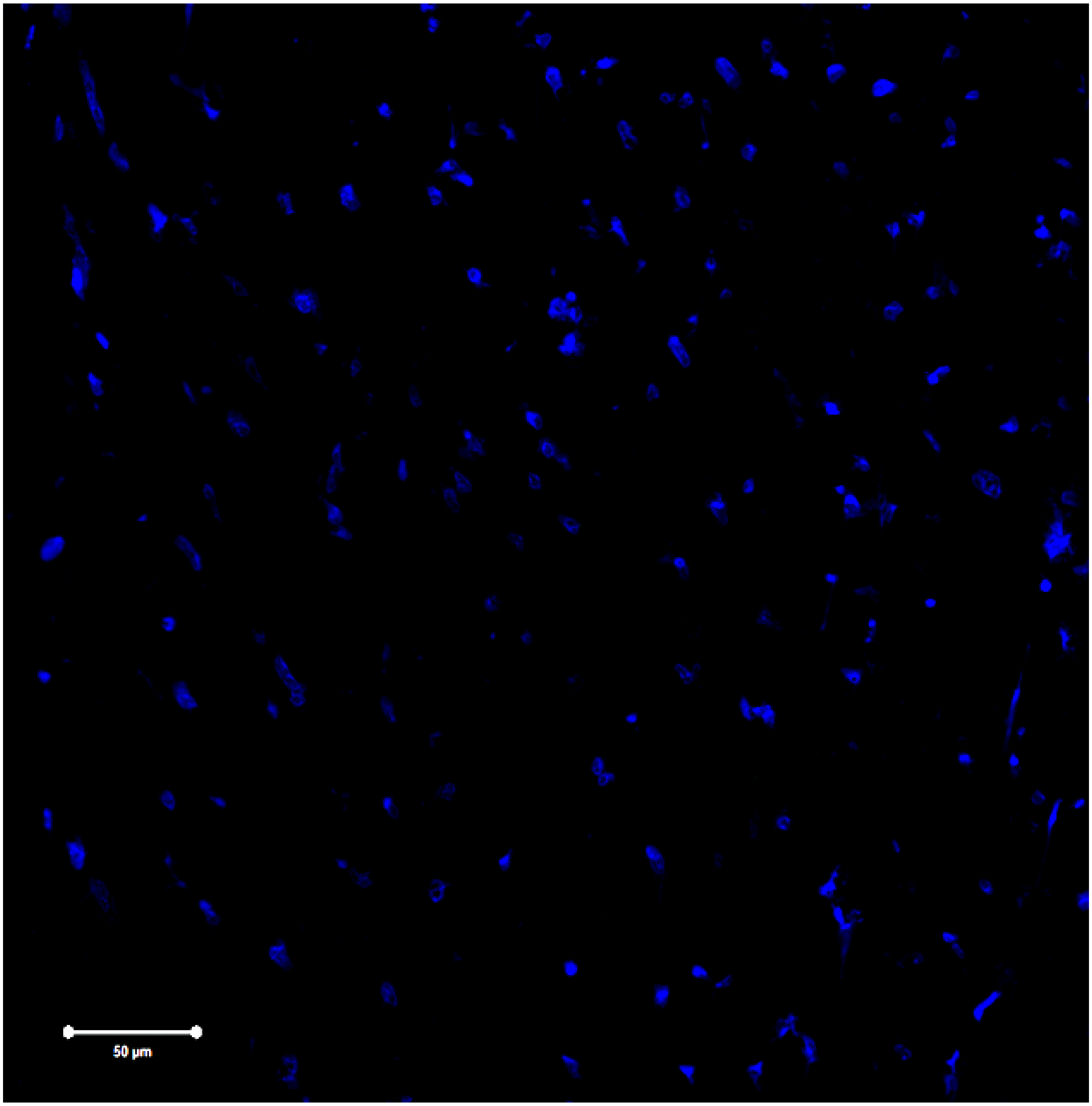

Supplementary Figure 14. High resolution version of Figure 4G (Control Sheep – CD14 Staining)

Supplementary Table 1. Antibodies used to detect progenitor marker expression via flow cytometry

Antibody	Manufacturer	Isotype	Conc.	Species	Clone	Cat. No.	Lot No.
Mesp1-DyLight 405	Novus Biologicals	IgG	0.8mg/mL	Rabbit	2030B	FAB9219V	CKNQ01-1-050317-V
Isotype-DyLight405	Novus Biologicals	IgG	1.05mg/mL	Rabbit	NBP2-36463V	NBP2-36463V	31908-050317-V
Islet1	Abcam	IgG1	0.83mg/mL	Mouse	1H9	ab86472	GR273015-3
Fluorescein Labeling Kit	Novus Biologicals	n/a		n/a	10934	707-0030	1BS3384
Isotype-FITC	BioLegend	IgG1 kappa	0.5mg/mL	Mouse	MOPC-21	400107	B199152
PDGFRα-PE	BioLegend	IgG1 kappa	100ug/mL	Mouse	16A1	323505	B192368
Isotype-PE	BioLegend	IgG1 kappa	200ug/mL	Mouse	MOPC-21	400113	B214532
CXCR4-PE/Vio770	Miltenyi Biotec	IgG1	82.5ug/mL	n/a	REA649	130-109-887	5170503056
REA CtRl-PE/Vio770	Miltenyi Biotec	IgG1	20ug/mL	n/a	REA293	130-104-616	5170201559
cKit-DyLight650	Novus Biologicals	IgG2B kappa	500ug/mL	Mouse	2B8	NB100-77477C	B147020-A
Isotype-AlexaFluor 647	R&D Systems	IgG2B	10ug/mL	Rat	141945	IC013R	AEIU0114121
SSEA1-APC/Vio770	Miltenyi Biotec	IgG1	8.25ug/mL	n/a	REA321	130-104-992	5161207356
REA Ctrl-APC/Vio770	Miltenyi Biotec	IgG1	30ug/mL	n/a	REA293	130-104-618	5170201560

Supplementary Table 2. Antibodies used in immunohistochemistry experiments

Antibody	Manufacturer	Isotype	Dilution	Species	Clone	Cat. No.	Lot No.
Ki-67	BioLegend	IgG1	1:200	Mouse	Ki-67	350507	B167963
Secondary AlexaFluor 633	Molecular Probes	IgG	1:200	Goat	Polyclonal	A21052	1622583
Isl1	Abcam	IgG1	1:100	Mouse	1H9	ab86472	GR141006-3
Secondary AlexaFluor 633	Molecular Probes	IgG	1:200	Goat	Polyclonal	A21052	1622583
TropI	Millipore	IgG2b	1:100	Mouse	C5	MAB1691	2536827
Secondary AlexaFluor 633	Molecular Probes	IgG	1:200	Goat	Polyclonal	A21052	1622583
vWF	Dako	Ig	1:200	Rabbit	Polyclonal	A0082	2061542
Secondary AlexaFluor 647	Abcam	IgG	1:200	Donkey	Polyclonal	ab150075	GR114884-1
CD14	Abcam	IgG	1:75	Mouse	2Q1233	ab63319	GR275025-3
Secondary AlexaFluor 555	Life Technologies	IgG	1:200	Goat	Polyclonal	A21422	1608465
Cxn43/GJA1	Abcam	IgG1	1:100	Mouse	4E6.2	ab79010	GR167778-1
Secondary AlexaFluor 633	Molecular Probes	IgG	1:500	Goat	Polyclonal	A21052	1622583

Table 3. **Primer pair sequences used in RT-PCR experiments**

Human Primer Pairs		
**Gene**	**Forward Sequence (5' to 3')**	**Reverse Sequence (5' to 3')**
ACTA2	AGCTTTCAGCTTCCCTGAACA	TACAGAGCCCAGAGCCATTG
ACTIN	TTTGAATGATGAGCCTTCGTCCCC	GTCTCAAGTCAGTGTACAGGTAAGC
MESP1	TAGGCCTCAGCGAGGAGAGT	TCCCTTGTCACTTGGGCTCC
MLC2V	GGTGCTGAAGGCTGATTACGTT	TATTGGAACATGGCCTCTGGAT
MYH11	CAAATACGCGGATGAGAGGGA	CTCATGGACGTTCTTGCCCA
PECAM1	AACGGAAGGCTCCCTTGATG	TAAGAACCGGCAGCTTAGCC
TNNT2	GTGGGAAGAGGCAGACTGAG	ATAGATGCTCTGCCACAGC
VWF	ACACCTGCATTTGCCGAAAC	ATGCGGAGGTCACCTTTCAG
TBX3	TGGCCTACCATCCGTTCCTA	GGACATCCACTGTTCCCCAG
TBX5	CTCAGTCCCCCGGAACAAC	CACGTACCTCCCAGCTCAAG
TBX18	GGTGGCAGGTAATGCTGACT	ACTTGCATTGCCTTGCTTGG
SHOX2	CTTACGGCGTTCGTCTCCAA	GACACCTCCGTCAGTCGC
HCN4	CAGCCTCTTACGCCTGTTAC	CCAGGAGTTGTTCACCATGTTG
GJC1	GAGGTGGAGGAGAGGCGAG	CCGAGCTGCCTTCTTGTCTG
ISL1	CACAAGCGTCTCGGGATTGTGTTT	AGTGGCAAGTCTTCCGACAA
FOXA2	GGAACACCACTACGCCTTCAA	AGTGCATCACCTGTTCGTAGG

Ovine Primer Pairs		
**Gene**	**Forward Sequence (5' to 3')**	**Reverse Sequence (5' to 3')**
ACTIN	CGAGATGAGATTGGCCTCGT	CATGGTTGCTAAGGGCAGGA
ISL1	GTGCAGCATCGGCTTCAGCAAAAA	CCTCCCGCAGCGCGAACTCAT
C-KIT	GGCATATCCCAAACCTGAACACCGA	CCCTGCGGCCACGCACTGTA
PIK3CA	AACAATGCCTCCACGACCAT	TCACGGTTGCCTACTGGTTC
CCND1	TCGAGCACTTCCTCTCCAAAA	GTTTGCGGATGATCTGCTTGT
JUN	AGCATGACCCTGAATCTGGC	GATATGCCCGTTGCTGGACT
MYC	CGCATCAGCACAATTACGCA	ACTCTGGGATCTGGTCACGA
RELA	GCGAGAGGAGCACAGATACC	GGGGTTGTTGTTGGTCTGGA
NOTCH1	GATCGAGGAGGCGTGCGAGC	CTTCCGCACGCTGGCAGTCA
NOTCH2	GATGGCCTGGGTACCTACCGCT	GCAGCGACAACTGTAGCCTCCAA
SOD2	ACCACGCGGCCTACGTGAAC	AGAAAGCCGAGTGTTTCCCTT
HMOX1	CAAGCGCTATGTTCAGCGAC	GTGTGAGGACCCATCGCAG
TNFAIP3	TCGGGAAGGAGCAGAGATGA	TTCTGGGGACCATGTGCTTC
BCL2	TGCACCTGACGCCCTTCAC	AGACAGCCAGGAGAAATCAAACAG
IGF	GACAGGAATCGTGGATGAGTG	AACAGGTAACTCGTGACGAGC

SDF1A	CCTTGCCGATTCTTTGAGAG	GGTCAATGCACACTTGCCTA
HGF	TGCACAATTCCTGAAAAGACC	CGGACAAAAATACCAGGACG
VEGF	TGCTCTCTTGGGTGCATTGG	GAAGCTGCGCTGGTAGACAT
CXCR4	AAGGCTATCAGAAGCGCAAG	GAGTCGATGCTGATCCCAAT
IL10	TTTTCCCTGACTGCCCTCTA	GCTCCCTGGTTTCTCTTCCT
TGFB	CAATTCCTGGCGCTACCTCA	GGTTCATGCCGTGAATGGTG
YAP1	GCACCTTCGACAGTCTTCCT	TTCTCTGGTTCATGGCAAAACG
AGRIN	CATCCATTATAAGCCTCAAGGGC	GCCAAAGCCACACAGCATTC
FOXA2	GGAACACCACTACGCCTTCAA	AGTGCATCACCTGTTCGTAGG
PTGS2	TAACACGCTCTACCACTGGC	GATTCCTACGACCAGCGACC
PTGER4	ATCGCCACCTACTTGAAGGG	GCTCGATACTCATCGCACA
BAX	TCTGAGCAGATCATGAAGACAGG	CGCCACTCGGAAAAAGACCT

Table 4. Antibodies used for western blot

Antibody	Species	Antibody Dilution	Catalog No.	Manufacturer
HCN	Mouse	1:10	ab85023	Abcam
AKT	Rabbit	1:50	4691S	Cell Signaling Technology
p-AKT (Ser 473)	Rabbit	1:10	4060S	Cell Signaling Technology
beta-Actin	Mouse	1:50	8H10D10	Cell Signaling Technology

Table 5. **Antibodies used for directed differentiation**

Antibody	Manufacturer	Isotype	Conc.	Species	Clone	Cat. No.	Lot No.
TropT-PE	Miltenyi Biotec	IgG1	10 µg/mL	n/a	REA400	130-106-746	5160426814
Isotype-PE	BD Pharmigen	IgG1 kappa	0.5 mg/mL	Mouse	MOPC-21	556650	19587
vWF-AlexaFluor657	Novus Biologicals	IgG1 kappa	0.75 mg/mL	Mouse	3E2D10	NBP2-34535AF647	7450-1PABX160519120816-AF647
Isotype-AlexaFluor647	R&D Systems	IgG2B	10 µg/mL	Rat	141945	IC013R	AEIU0114121
αSMA-PerCP	Novus Biologicals	IgG2a kappa	0.4 mg/mL	Mouse	1A4/asm1	NBP2-34522PCP	59-1PABX170619-090617-PCP
Isotype-PerCP	Miltenyi Biotec	IgG1	55 µg/mL	Mouse	IS5-21F5	130-094-968	5110906188

Supplementary Table 6. Figure 1C gene list

ADRA1D	**Calcium Regulation**	NRG2	**ERBB4 Signaling**
ADCY4		FOXO1	
ARRB1		TGFA	
GJA4		CAMK2A	
ADCY5		AREG	
ATP1B2		EREG	
PRKAR2B		TCF7L1	
CACNA1B		PPP2R2B	**WNT Signaling**
GJB4		WNT3A	
SLCBA3		FOXD3	
RGS16		PRKCH	
GNAO1		WNT7A	
KCNJ5		FRAT1	
PRKCH		LEF1	
RGS14		FZD9	
CACNA1C		WNT4	
RGS3		FZD3	
KCNB1		FZD4	
ADRB1		PPM1J	
ARRB2		SOX2	
CHRM3		MAPK10	
CAMK2A		CD44	
CAMK2D		WNT7B	
RGS2		FZD7	
GJA5		WNT5B	
SLC8A1		WNT15	
SOX1	**Cardiac Progenitor Differentiation**	CCN2	
NKX2-5		WNT5A	
KDR		TEAD2	**Mesodermal Commitment**
GATA4		VAV3	
MESP1		TBX1	
TNNI3		TCF7L1	
SCN5A		ZIC2	
T		RARB	
WNT3A		BMP7	
BMP4		WNT3A	
NOG		BMP4	
PAX6		NOG	
KIT		DLL1	
SOX17		PAX6	
CXCR4		LEF1	
NOTCH1		SOX17	
SOX2		CHRD	
FGF2		ACVR2B	
IRX4		FZD4	
PDGFRA		SOX2	
NKX2-5	**Heart Development**	DNMT3B	
TBX1		FOXC2	
GATA4		WDFY2	
BMP4		HMGA2	
SHH		TBX3	
NFATC1		BMPR1A	
HEY1		PARP8	
NOTCH1			
IRX4			
FOXC2			
BMPR1A			
TBX2			

Supplementary Table 7. Quantification of percent labeled sheep cells: Stained vs. Controls

	Mesp1	Isl1	PDGFRα	CXCR4	c-Kit	SSEA1
**Marker Positive**	**73.0**	**83.3**	**80.6**	**30.0**	**63.0**	**18.3**
**Negative Control**	**0**	**0.04**	**0.24**	**3.0**	**2.63**	**3.0**
**Isotype**	**4.91**	**4.53**	**4.69**	**6.0**	**1.51**	**4.91**

Supplementary Table 8. Quantification of percent labeled human cells: Stained vs. Controls

	Mesp1	Isl1	PDGFRα	CXCR4	c-Kit	SSEA1
**Marker Positive**	74.6	92.9	92.4	36.8	82.1	42.8
**Negative Control**	0	0.02	1.95	0.85	2.83	2.95
**Isotype**	4.96	4.37	3.13	4.91	2.02	3.22

Supplementary Table 9. Quantification of geometric mean of sheep cells: Stained vs. Controls

	Mesp1	Isl1	PDGFRα	CXCR4	c-Kit	SSEA1
**Marker Positive**	2622	545	48.7	23.9	2.12	2.15
**Negative Control**	23.6	17.2	4.17	7.27	0.45	1.36
**Fold Change**	111.1	31.7	11.7	3.3	4.7	1.6

Supplementary Table 10. Quantification of geometric mean of human cells: Stained vs. Controls

	Mesp1	Isl1	PDGFRα	CXCR4	c-Kit	SSEA1
**Marker Positive**	35.4	18.2	2.39	2.79	5.7	2.34
**Negative Control**	3.17	0.87	0.4	0.63	1.11	1.03
**Fold Change**	11.2	20.9	6.0	4.4	5.1	2.3

Supplementary Table 11. Cell-Treated vs Non-Infarct analysis of *BAX* expression.

Log2 FC	p-value	P adjusted	Gene Name
0.313249	0.740425	0.939687	BAX
